# Cognotoxemia: endotoxemia and gender predict changes in working memory performance in healthy adults

**DOI:** 10.3389/fnins.2024.1453325

**Published:** 2024-11-06

**Authors:** Sally C. McDonnell, Jennifer E. Graham-Engeland, Martin J. Sliwinski, Christopher G. Engeland, Erik L. Knight

**Affiliations:** ^1^Department of Psychology and Neuroscience, University of Colorado Boulder, Boulder, CO, United States; ^2^Department of Biobehavioral Health, Pennsylvania State University, University Park, PA, United States; ^3^Center for Healthy Aging, Pennsylvania State University, University Park, PA, United States; ^4^Department of Human Development and Family Studies, Pennsylvania State University, University Park, PA, United States; ^5^Ross and Carol Nese College of Nursing, Pennsylvania State University, University Park, PA, United States

**Keywords:** cognition, endotoxemia, working memory, gut-brain axis, biomarker

## Abstract

**Introduction:**

Examining the contribution of peripheral systems to cognitive function under healthy circumstances may improve our understanding of the systems that confer risk or resilience in diseased states. Endotoxemia—a pro-inflammatory response to the translocation of bacteria that reside in the gut on other sources (e.g., respiratory tract; infection) into the blood—was hypothesized to relate to worsened cognitive functioning. Gender was explored as a moderator.

**Methods:**

A sample of 162 healthy adults (25–65 years old) provided plasma, from which a measure of endotoxemia was determined [i.e., the ratio of lipopolysaccharide binding protein (LBP) to soluble cluster of differentiation 14 receptors (sCD14)]. Participants performed an array of laboratory and ambulatory cognitive tasks at three timepoints, each separated by 9 months. Two sets of multilevel models were used: Prospective models, linking endotoxemia at baseline with changes in cognition across time, and coupling models, which examine correlations of endotoxemia with cognition across time.

**Results:**

A prospective model indicated lower levels of endotoxemia at baseline predicted improvements in working memory across the three timepoints; higher levels were associated with no change in cognitive performance. Gender was not found to modulate this finding. Interestingly, a coupling analysis of endotoxemia and gender across time showed that in men, those with higher endotoxemia performed better at the working memory task overall; in women, working memory performance was similar regardless of endotoxemia level.

**Conclusion:**

This work provides initial evidence that endotoxemia may be associated with a dampening of improvement in working memory, improvement consistent with practice effects, which should be expected in a sample of healthy, relatively young adults. The findings also provide preliminary evidence that, at least for men, higher degrees of endotoxemia are not inherently negative, and may link with short term positive outcomes for working memory.

## 1 Introduction

Understanding the contributing factors to age-related cognitive decline is increasingly imperative as the national population’s median age continues to rise (U.S. Census Bureau, 2023). More people are being affected by dementia, with nearly 10 million new cases diagnosed worldwide every year (World Health Organization, 2023). Thus, it is important to identify possible biological contributors to various forms of cognitive decline earlier in life.

In recent years, interest has grown surrounding how the gut may impact cognitive health (Yarandi et al., 2016; Proctor et al., 2017; Carabotti et al., 2015). The dominant theory to date proposes that there is a bidirectional relationship between the gut and brain, which may allow bioactive metabolites and microbiome-induced inflammation to affect activity of the central nervous system, including mood states and behavioral responses to stress (Yarandi et al., 2016; Rea et al., 2016; Donoso et al., 2023). Prior research has explored gut-microbiome composition, steroids, C-reactive protein (CRP), and various cytokines [including interleukin (IL)-6, tumor necrosis factor (TNF)-ɑ, and others], linking these compounds with alterations in brain morphology (Marsland et al., 2015) and various forms of cognitive change (Liu et al., 2022; Yarandi et al., 2016; Tillisch et al., 2013; McAfoose and Baune, 2009; Misiak et al., 2018). The present work seeks to explore the relationship between another marker of gut-associated inflammation, endotoxemia—the (non-clinical) pro-inflammatory response to the release of endotoxins from the gut into the blood—and cognition.

Gut-related processes may contribute to cognition via several routes. Long-term alterations in gut microbiota composition may potentially lead to changes in gut permeability, promoting “leakiness” and bacterial translocation into the blood. Such changes, in turn, have been linked to increases in immune-mediated inflammatory responses and changes in mood (Kiecolt-Glaser et al., 2018) and have been associated with cognitive functioning (Madison et al., 2020). This emerging endotoxemia-to-immune-to-cognition pathway is indicative of the need to look upstream from inflammatory markers (e.g., cytokines) to better understand how natural, biologically-necessary peripheral processes may impact the brain. Exploring endotoxemia as one of many precursors to an inflammatory immune response allows us to examine a distinct process and possible intervention target that links the gut and brain.

Endotoxins (such as lipopolysaccharides [LPS]) are large molecules found on the outer membrane of gram-negative bacteria and are potent stimulators of the immune system (André et al., 2019). Endotoxin molecules are largely released when bacteria die and primarily emanate from bacteria in the gut, though they can also originate from bacteria on mucosal linings, at the site of local infection, and within the respiratory tract (André et al., 2019). From the gut, endotoxins and other microbes translocate into blood at low levels through tight junctions in the gastrointestinal tract, a normal and natural process (André et al., 2019). However, high levels of intestinal permeability are harmful and can signal pathogenesis (Odenwald and Turner, 2013). Once in the circulation, endotoxins are bound by lipopolysaccharide binding protein (LBP), which acts as a chaperone protein to direct endotoxins to cluster of differentiation-14 (CD14) receptors (Knight et al., 2020). There are two main types of CD14 receptors that bind endotoxins, membrane-bound (mCD14) and soluble (sCD14) (André et al., 2019). mCD14 is present on the cell-surface of various immune cells (such as macrophages and monocytes) and generally leads to a cascade of proinflammatory effects (Pollack et al., 1997). sCD14, which is derived from the cleavage of mCD14 in response to ongoing exposure to endotoxins and other pro-inflammatory activity, serves to attenuate the body’s response by transporting LPS/endotoxins to high density lipoprotein (HDL) for eventual breakdown in the liver (Wurfel et al., 1995; Pajkrt et al., 1996). Hence, the sCD14 pathway can be considered non-inflammatory; HDL sequesters endotoxins that would otherwise lead to further inflammatory mCD14 response (Kitchens and Thompson, 2005; Knight et al., 2020). Both LBP and sCD14 are present in plasma and can be used to approximate levels of endotoxemia, a state indexed by a higher ratio of LBP:sCD14, which is indicative of a predisposition to higher pro-inflammatory responses to endotoxins (Laugerette et al., 2014; Knight et al., 2020).

Previous research has linked endotoxemia to differences in attention and executive function. A higher LBP:sCD14 ratio was associated with impaired performance on Continuous Performance Tasks (CPTs) in women; deficits included worse response time and impaired shifting or sustaining of attention (Madison et al., 2020). Administration of an antibiotic that is thought to reduce endotoxemia (Rifaximin) has been shown to rescue deficits in working memory (N-Back task) and inhibitory control in mixed-gender clinical populations (Ahluwalia et al., 2014). Additionally, various markers of endotoxemia have been associated with slower cognitive processing speed in men, though this research is limited due to small sample sizes and potential confounding clinical conditions, such as alcohol use disorder and HIV (Monnig et al., 2017). Even fewer studies have examined longitudinal changes in cognitive functioning in relation to LBP, sCD14, or their ratio. Preliminary findings in a small sample of mixed-gender obese individuals indicate that higher baseline LBP levels predict poorer working memory 2 years later, and that changes in working memory correlate negatively with changes in LBP (Moreno-Navarrete et al., 2017). No work, to our knowledge, has examined longitudinal cognitive change in relation to the LBP:sCD14 ratio or how this relationship may differ between men and women.

Despite existing literature which proposes that bacterial translocation from the gut may worsen executive function, attention, and other measures of cognition (Madison et al., 2020; Ahluwalia et al., 2014; Monnig et al., 2017), some work has indicated an opposite pattern. For example, circulating levels of LPS have been correlated positively with performance on the Trail Making Test B task, a measure of executive function (Monnig et al., 2017). However, this finding should be interpreted with caution, as the physiological response to bacterial translocation (e.g., indexed by LBP and sCD14) is typically regarded as more relevant to clinical cognitive outcomes than the microbial levels themselves. LBP and sCD14 were not associated with improved executive function in this same study (Monnig et al., 2017). It is important to note that these mixed findings could potentially be due to measurement difficulties with LPS, which has a short half-life (Yao et al., 2016) and is challenging to measure in humans (Novitsky, 1998). LBP is a much more stable molecule, with a half-life of several days (Turgunov et al., 2024) and has moderate test–retest reliability over several months (Citronberg et al., 2016). The LBP:sCD14 ratio is a comparatively more stable and reliable measure than LPS, and so may be a better indicator of associations between endotoxemia and cognition.

In addition to these somewhat mixed findings related to executive function, there is also mixed evidence that endotoxins and endotoxemia are involved in neurodegeneration. In rats, a single intraperitoneal administration of LPS was found to increase neuroinflammation, *β*-amyloid plaques, and p-tau levels in the brain (Wang et al., 2018). However, LPS exposure may also be neuroprotective, priming microglia to a more anti-inflammatory response (Mizobuchi and Soma, 2021). Both rats in a rodent model of Alzheimer’s Disease (AD) and humans diagnosed with AD have been found to exhibit elevated blood levels of endotoxins (Han et al., 2016). Elevated levels of plasma endotoxins (achieved either through LPS-administration or disease state) may signal heightened blood–brain-barrier permeability or directly increase it, with downstream inflammatory consequences that can accelerate neurodegeneration in conditions like AD and Parkinson’s Disease (Brown, 2019). It follows that the degree and timeline of exposure to endotoxins, as well as the characteristics of the immune response to it, are likely important determinants of the risk or resilience conferred in disease states. Endotoxemia’s association with cognition may mirror the results seen with administration of endotoxins (LPS), in terms of having paradoxically positive and negative effects on cognition.

In summary, previous studies indicate some relationships between endotoxemia, cognition, and neurodegeneration, but the extent and direction of these connections is still largely unknown. The purpose of this study is to investigate the degree to which endotoxemia is associated with various aspects of cognition, including episodic memory, working memory, and reasoning ability in a non-clinical, healthy, adult sample. Cognition was operationalized with a variety of laboratory tasks and ecologically assessed ambulatory tasks (7 in total). Such a wide array of cognitive tasks will allow us to gain a better understanding of endotoxemia’s associations with cognition in general, and with specific aspects of cognition. It was hypothesized that higher LBP:sCD14 would correlate with worsened performance on working memory tasks, based on prior work that has studied attentional effects (Madison et al., 2020). Worsened performance was hypothesized to include both lower scores on working memory measures for those with higher levels of LBP:sCD14 within a given wave and a lack of improvement (i.e., lack of practice effects) on these measures across time that would typically be expected in a healthy adult sample. We also explored the extent to which endotoxemia correlates with processing speed and episodic memory. In addition, previous work indicates that endotoxemia drives gender differences in the link between stimulated inflammation and depressive symptoms (Knight et al., 2020). Hence, we explored whether endotoxemia would be more strongly associated with cognition for men compared to women, with follow-up analyses exploring the effects of self-reported menopause on these associations.

## 2 Methods

### 2.1 Design

This project used a subset of data from a larger study, The Effects of Stress on Cognitive Aging, Physiology and Emotion (ESCAPE) Project. The ESCAPE Project has primarily explored how psychological stress responses can mediate the relationship between stress and broad-scale cognitive function. The original study utilized a prospective, longitudinal design, with data collected in a series of waves (at baseline [Wave 1], 9 months [Wave 2], and 18 months [Wave 3]).^1^ Within each wave, ecological data was also collected multiple times per day via smartphone in 14-day measurement bursts. For more details, see Scott et al., 2015.

### 2.2 Procedure

After informed consent was obtained, participants were mailed an initial survey which collected demographic and other baseline information. They attended an initial lab visit, where they received training on how to perform ecological momentary assessments on a study-provided smartphone. At the beginning of each wave, participants came into the lab and performed the cognitive assessments described later in this section. For the next 14 days, the participants’ smartphones would beep 5 times per day, prompting them to complete a survey and three ambulatory cognitive tasks. At the end of this two-week period, participants returned to the lab and completed a blood draw after 12 h of fasting. There were three waves of data collection (baseline, 9 months, 18 months) in which participants would follow this protocol. Compensation was based on completion of each wave. A maximum of $160 was given to participants for each wave based on their degree of compliance with the study protocol.

### 2.3 Participants

Participants were recruited for the ESCAPE Project using systematic probability sampling via New York Registered Voter Lists. Potential participants were sent recruitment letters that described the study goals, and a follow-up telephone call was used to enroll participants in the study. To be eligible for the study, participants had to be 25–65 years old, ambulatory, fluent in English, without visual impairment, and live in Bronx County. Inability to answer smartphone-based survey questions throughout the day was the main exclusion criteria. This approach is naturalistic by design and was not intended as a clinical sample that recruited balanced numbers of participants based on age, race, gut or immune functioning, or some other characteristic.

A sub-sample of the original study was analyzed for this project, including 162 participants for whom the necessary blood samples had been collected. This subset of participants was chosen using these criteria: Participants had no psychiatric conditions outside of depression, were not currently taking immunosuppressive drugs, and had no history of inflammatory illnesses like autoimmune conditions, diabetes, HIV, kidney/liver disease, or cancer. Thus, this is a physically “healthy” subset of the original ESCAPE sample. For more information, see Knight et al., 2020.

Participants were aged 25–65 years (*M* = 44.44, SD = 11.16). By self-report, 70% identified as Black and 22% identified as Hispanic/Latinx, matching the diversity of the geographic area from which it was recruited (Bronx, NY) and making the sample generalizable to a broader population than what has been typical in biomedical or clinical research (Cooke Bailey et al., 2020; Polo et al., 2019). Participants were asked to report their sex/gender identity; the term “gender” (and not “sex”) is used in this report to better encompass the sum total of biopsychosocial differences in immune functioning between men and women (Knight et al., 2020; Darnall and Suarez, 2009). Of the total sample, 67% identified as female and 33% identified as male. Of the 109 women included in this study, 36 identified as post-menopausal (33% of women). See Table 1 for more information about participant demographics.

**Table 1:** Demographic information.

Age	
Mean (SD)	44.44 (11.16) years
Range	25–65 years old
Race	
Black (%)	113 (70%)
Non-Black (%)	49 (30%)
Ethnicity	
Hispanic (%)	35 (22%)
Non-Hispanic (%)	127 (78%)
Gender	
Female (%)	109 (67%)
Male (%)	53 (33%)
Education	
High School or Less (%)	41 (25%)
Some College (%)	45 (28%)
College Degree (%)	48 (30%)
Graduate or Professional Degree (%)	28 (17%)
Total (n)	162

### 2.4 Measures

A variety of cognitive measures were employed in this study to get a holistic understanding of how endotoxemia may relate to cognition. A majority of cognitive tasks were administered in a research clinic by a trained technician; three cognitive tasks were administered using an intensive, ecological momentary assessment approach on smart phones to gauge ambulatory cognitive functioning. Laboratory cognitive tasks were identical across the three timepoints, allowing us to detect longitudinal practice effects in these measures.

#### 2.4.1 Working memory

There were three standardized laboratory-based working memory tasks utilized in the ESCAPE protocol to assess working memory capacity of the participants: Operation Span, Counting Span, and Backward Letter Span.

Operation Span was collected in this study by instructing participants to remember letters that they saw on screen (Turner and Engle, 1989). In between letter presentations, they were also instructed to verify the accuracy of a math equation. Participants were told to report whether the equation was correct aloud with either a “yes” or “no,” as well as to read each letter aloud. A “strict” score was calculated from participant performance (Conway et al., 2005). This strict score is an all-or-nothing approach; participants received either full credit for answering the trial entirely correct or no credit. Performance on each question was summed to create an overall score for each participant and recorded by hand.

In the Counting Span task (Case et al., 1982) participants were shown a series of images that included dark blue circles, dark blue squares, and light blue circles on a computer screen. They were instructed to count the number of dark blue circles aloud. At the end, they were instructed to repeat the final number of dark blue circles aloud. Participants would continue this for some time before being asked to repeat the totals from each slide in order (Pluck et al., 2016). Overall scores on the measure were calculated by summing the number of sequences participants were able to correctly recall in the proper order. Participants did not receive partial credit for remembering part of the sequence or recalling the sequence out of order. Scores were again computed and recorded by hand.

For the Backward Letter Span (BLS) task (Colom et al., 2005) participants were informed by the technician that they would see some letters. They were instructed to remember the letters and be prepared to recall them in reverse order. After all the letters were presented, participants were then prompted to recall them in the reverse order as instructed before the task. Again, participants were scored “strictly.” Strict scores allocated no partial credit for recalling some of the sequence in the right order. Scores on each question of the task were summed for each participant to compute an overall score and recorded by hand.

Performances on the three working memory tasks were highly correlated (*r* > 0.5; Supplementary Table S1) and so they were combined into a single, composite score of working memory (Cronbach’s *α* = 0.72). To do so, strictly scored working memory outcomes (OS, CS, BLS) were z-scored at each wave, using Wave 1[baseline] means and standard deviations to produce the z-score. The three working memory z-scores in a given wave were then averaged for each participant. The principal analyses focused on the composite measure, with follow-up (Supplementary) analyses focusing on the individual tests.

#### 2.4.2 Auditory verbal learning test

The Auditory Verbal Learning Test (AVLT) is a measure of episodic memory (Binder et al., 1993). Participants were informed that they would see a list of words presented on a screen in front of them. They were instructed to study the words for 1 min. After the minute was up, the screen changed to three question marks. Participants were told that the presence of these three question marks was a cue for them to recall all the words from the list that they could. This task was done in the research clinic and overseen by a technician. The total number of words correctly recalled by the participant (out of 15 presented words) was calculated by the technician. Participants earned one point per correct word, and these point values were utilized in this study to approximate episodic memory performance.

#### 2.4.3 Ambulatory cognitive data

Participants completed three ambulatory cognitive tasks using a study-issued smartphone (Scott et al., 2015). Participants were beeped to begin the cognitive testing at five time points scheduled quasi-randomly to cover the entire waking day (participants also responded to several questionnaire items, which are not analyzed here; see Scott et al., 2015 for more details). The tasks are described in the fixed order in which participants completed them at each beep.

The first task was a measure of processing speed and required participants to decide which symbol pairs at the bottom of their screen matched those at the top of the screen. Processing speed was indexed by measuring mean reaction time across all correct trials within a wave. Reaction times were log-transformed prior to analysis. The second task assessed spatial working memory. Participants were asked to recall the locations of three red dots that were presented for 3 s and followed by a distractor image that was presented for 8 s. Spatial working memory scores were calculated using the mean Euclidian error distance for each dot-location (i.e., how far participant responses were from the actual locations) across all trials in a wave. Scores represent an error score (higher values indicate worse performance) and were log-transformed prior to analysis. Finally, verbal working memory was measured with a modified N-Back task (Verhaeghen and Basak, 2005). Three playing cards slid from a box on one side of the screen to a box on the other side. Participants were asked to compare whether the cards in the two boxes were the same when presented with the cards face-up (0-back) and when the previous two cards were face-down (2-back). Visual working memory capacity was operationalized by computing the mean reaction time of all correct 2-back trials within a test (i.e., at a given beep). An inverse efficiency score (IES) was then calculated by dividing mean reaction time by the proportion of correct responses in that test. The IES was then averaged across all the N-Back tests within a wave. Higher values indicate worse performance (slower reaction time and/or worse accuracy). Each ambulatory cognitive assessment consisted of 16 symbol-searches, 2 spatial working memory trials, and 12 rounds of the 2-back.

#### 2.4.4 Bioassays

Endotoxemia was approximated by calculating the ratio of plasma levels of LBP and sCD14. In this paper, endotoxemia refers to a non-clinical state combining heightened exposure to LPS (indexed by LBP) and reduced reliance on non-inflammatory clearance (indexed by lower sCD14), which is indicative of a proinflammatory response to endotoxins. 5 mL of blood was collected by a certified phlebotomist between 7 a.m. and 11 a.m. and stored in sodium heparin tubes. After collection, blood was centrifuged at room temperature for 15 min at 3000 g. The supernatant was aliquoted and stored at −80°C. Levels of sCD14 and LBP were determined with commercial kits (LBP: sandwich immunoassay from Meso Scale Discovery, Rockville, MD; sCD14: ELISA from R&D Systems, Minneapolis, MN). To be detected, levels of LBP and sCD14 had to reach a minimum threshold of 0.038 ng/mL and 125 pg/mL, respectively. Inter-assay CVs were 11.5% (LBP) and 3.6% (sCD14) and intra-assay CVs were 11.6% (LBP) and 2.7% (sCD14) (Knight et al., 2020). All assays were performed in duplicate and were repeated for samples with CV > 15%. The ratios of LBP:sCD14 for each participant were computed and then standardized (z-scored) prior to entry into the model.

### 2.5 Statistical analyses

Two complementary, analytical approaches were used to examine the data. To examine the prospective relationship between endotoxemia and cognitive functioning, a cognitive outcome from a given wave was regressed on the interaction between Wave 1 (baseline) endotoxemia and time (i.e., wave number, which was contrast coded as a linear and quadratic effect). Covariates included gender, age, race, ethnicity, Wave 1 BMI, and education. Random intercepts and slopes of (linear) time per participant were included.

We also examined a multi-level coupling model to investigate the correlation of endotoxemia and cognition across waves (Marceau et al., 2015). A cognitive outcome from a given individual and wave was regressed on that individual’s endotoxemia value for that wave, controlling for time (linear and quadratic contrast of wave), gender, age, race, ethnicity, and education, with random slopes and intercepts of endotoxemia per participant. Whereas the prospective models described previously provide information on how endotoxemia at baseline predicts change in cognitive functioning over time, the coupling models determine the extent to which changes in endotoxemia correlate with changes in cognitive functioning. Follow-up analyses from either approach used simple slopes to unpack significant interactions (Preacher et al., 2006).

Each set of models was then repeated with gender included as a moderator (dummy coded; Male = 0, Female = 1). Menopause status was further explored as a moderator due to known effects of menopause on epithelial barrier function that may impact endotoxemia levels (Looijer-van Langen et al., 2011; El-Lakany et al., 2018). These analyses relied on a a two-level Helmert contrast code for gender (Level 1: Men = 2, Pre-menopausal women = −1, Post-menopausal women = −1; Level 2: Men = 0, Pre-menopausal women = 1 Post-menopausal women = −1), which compares differences between pre-versus post-menopausal women (controlling for gender differences) and between women versus men (controlling for menopausal status).

All statistical analyses were done in R (v4.3.1, R Core Team, 2023) using multilevel modeling with the lme4 (Bates et al., 2015) and lmerTest packages (Kuznetsova et al., 2017). All graphs (from either approach) rely on estimated marginal means derived from each model, with cognitive performance graphed at lower, mean or higher levels (lower/higher = ±1 standard deviation from the mean) of endotoxemia.

We conducted power analyses to examine the smallest effect we were 80% powered to detect in these secondary data analyses. The exact number of participants (and the exact number of men and women) were input into Monte Carlo simulations with endotoxemia simulated as a normally distributed variable across the sample. Random intercepts and slopes per participant were included with assumed covariance of 0 and variance of 0.5. The models were simulated 1,000 times at each effect size between *b* = 0.1 and *b* = 1 (weak to strong standardized effects sizes) in 0.02 increments. We examined the highest order interaction term in each sets of models (i.e., Time × Endotoxemia in the prospective model; Gender × Endotoxemia in the coupling model). Results from the prospective model simulation indicate the study was 80% powered to detect a time Time_Linear_ × Endotoxemia effect of *b* = 0.20. The coupling model simulations indicate the study was 80% powered to detect a cognitive outcome’s association with an endotoxemia × gender interaction at an effect of *b* = 0.3. Because these models were based on the makeup of the actual sample, the results indicate we are unlikely to experience issues related to unbalanced sample sizes (e.g., due to gender), which extend from doing basic science in diverse samples. Though these analyses were conducted after data collection occurred, they do not rely on the detected effect of the analyzed data; hence, they are not *post hoc*—the analyses do not attempt to infer population metrics from sample outcomes—and are unlikely susceptible to the bias inherent to *post-hoc* power analyses (Zhang et al., 2019). Instead, these analyses simulated the power to detect an array of effect sizes given the constraints of our sample size and sample composition in these secondary data analyses.

## 3 Results

### 3.1 Preliminary findings

Table 2 provides descriptive statistics of LBP, sCD14, and their ratio. Intraclass correlation of the LBP:sCD14 ratio was examined via a two-way random effects model with absolute agreement and multiple measurements, revealing good within-participant reliability in endotoxemia measurement across time [ICC(2,k) = 0.81]. There were no evident gender differences in the LBP:sCD14 ratio at baseline (*t*(120.51) = 0.59, CI [−0.219, 0.407], *p* = 0.554, Table 2) or overall, when examining the ratio across all available data (*B* = −0.01, CI [−0.27–0.26], *p* = 0.95, Supplementary Table S2). Though there is not a truly established “healthy” level of these biomarkers, levels of LBP, sCD14, and their ratio were comparable to what has been seen in other studies (Madison et al., 2020; Kiecolt-Glaser et al., 2018; Gonzalez-Quintela et al., 2013).

**Table 2:** Means (SDs) of LBP, sCD14, and their ratio.

	Wave 1			Wave 2			Wave 3		
	Women	Men	All	Women	Men	All	Women	Men	All
*LBP* (μg/mL)	2.99 (1.34)	2.75 (1.37)	2.91 (1.35)	4.13 (2.06)	4.50 (2.43)	4.24 (2.17)	3.78 (1.65)	5.25 (4.03)	4.23 (2.68)
*sCD14* (μg/mL)	1.48 (0.297)	1.38 (0.347)	1.45 (0.316)	1.54 (0.408)	1.51 (0.365)	1.53 (0.394)	1.34 (0.245)	1.45 (0.506)	1.38 (0.347)
*LBP:sCD14*	2.07 (0.982)	1.99 (0.826)	2.04 (0.932)	2.67 (1.07)	3.05 (1.43)	2.79 (1.12)	2.84 (1.18)	3.53 (1.55)	3.05 (1.34)

For descriptive statistics of the cognitive tasks at baseline (Wave 1), see Table 3. All cognitive tasks were scored on a points system, excluding the symbol search task (reaction time in msec), the N-Back (IES, or the ratio of reaction time to percent accuracy; reported in msec), and the spatial working memory (Euclidian distance error scores) (Sliwinski et al., 2018). In Wave 1, gender differences were observed on the Spatial Working Memory task, in which women performed significantly worse (i.e., more errors, higher Euclidian distance error score) than men. Across time, differences in performance were observed on the Operation Span task, with men again performing better than women (OS, *B* = 3.14, [0.95–5.34], *t*(144.96) = 2.82, *p* = 0.005, Supplementary Tables S3, S4).

**Table 3:** Means (SDs) of cognitive task performance in Wave 1.

Task type	Task	Women	Men	All
*Working memory*	Operation Span	10.13 (6.78)	12.52 (7.77)	10.90 (7.17)
	Counting Span	20.71 (13.40)	21.96 (13.52)	21.13 (13.41)
	Backward Letter Span	8.00 (7.47)	8.43 (6.41)	8.14 (7.12)
*Auditory/Verbal*	AVLT	8.22 (2.45)	8.11 (2.53)	8.19 (2.47)
*Ambulatory*	Symbol Search	7.85 (0.24)	7.85 (0.25)	7.85 (0.25)
	N-Back	0.82 (0.12)	0.84 (0.13)	0.82 (0.12)
	N-Back (IES)	6.67 (0.33)	6.63 (0.33)	6.66 (0.33)
	**Spatial WM Error**	1.31 (0.46)	1.09 (0.50)	1.24 (0.49)

Bolded information indicates there was a significant gender difference between men and women. IES, Inverse Efficiency Score (ms).

### 3.2 Baseline endotoxemia prospectively predicts changes in working memory

**Figure 1:**
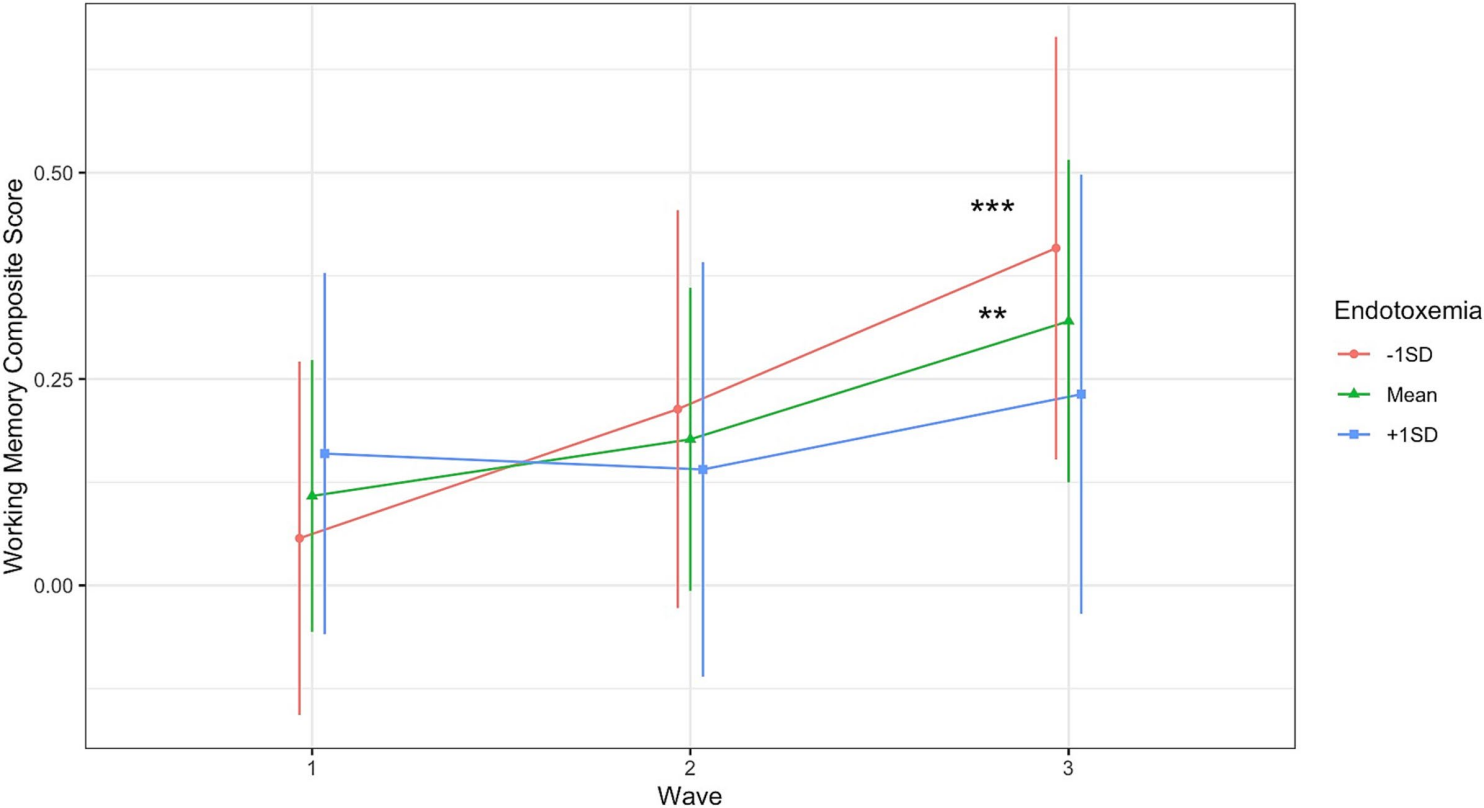
Endotoxemia at baseline prospectively predicted changes in composite working memory across time (Time_Linear_ × Endotoxemia, *B* = −0.10, [−0.19, −0.004], *t*(169.39) = −2.03, *p* = 0.044). Simple Slopes: **p < 0.005, ***p < 0.001.

Endotoxemia levels at baseline prospectively predicted changes in working memory across time (Time_Linear_ × Endotoxemia, *B* = −0.10, [−0.19, −0.004], *t*(169.39) = −2.03, *p* = 0.044; Figure 1; Table 4).^2^ Individuals with lower or moderate levels of endotoxemia demonstrated the expected improvement in (composite) working memory performance across the three measurements due to practice effects (simple slope at -1SD endotoxemia, *B* = 0.249, *t*(170.65) = 3.62, *p* < 0.001; at mean, *B* = 0.15, *t*(170.47) = 2.99, *p* = 0.003, Figure 1); individuals with higher endotoxemia levels demonstrated no gain in working memory across measurements (*B* = 0.051, *t*(169.20) = 0.717, *p* = 0.475, Figure 1). See Supplementary section for results with individual working memory tasks. This effect did not depend on gender, age, race, or education. No such effects of endotoxemia were evident for the other cognitive tasks (i.e., the AVLT and ambulatory measures) (Supplementary Tables S5, S6).

**Table 4:** Endotoxemia predicts prospective changes in composite working memory score.

	Composite working memory		
Predictors	Estimates	CI	*p*
(Intercept)	1.13	0.33–1.93	**0.006**
Time Linear	0.15	0.05–0.25	**0.003**
Time Quadratic	0.03	−0.06 to 0.12	0.527
Wave 1 LBP:sCD14	−0.02	−0.16 to 0.11	0.728
Age	−0.02	−0.03 to −0.01	**0.001**
Gender	0.24	−0.02 to 0.51	0.070
Race	−0.14	−0.45 to 0.17	0.389
Ethnicity	−0.24	−0.60 to 0.11	0.172
Education	0.23	−0.02 to 0.48	0.071
Wave 1 BMI	−0.00	−0.02 to 0.01	0.629
Time Linear × Wave 1 LBP:sCD14	−0.10	−0.19 to −0.00	**0.044**
Time Quadratic × Wave 1 LBP:sCD14	0.01	−0.08 to 0.11	0.761
Random effects			
σ^2^	0.22		
τ_00 Id_	0.52		
τ_11 Id.burst_L1_	0.00		
ρ_01 Id_	1.00		
N_Id_	162		
Observations	343		
Marginal *R*^2^/Conditional *R*^2^	0.268/NA		

Bolded values indicate statistical significance, *p* < 0.05.

### 3.3 The coupling of endotoxemia and working memory across time is moderated by gender

**Figure 2:**
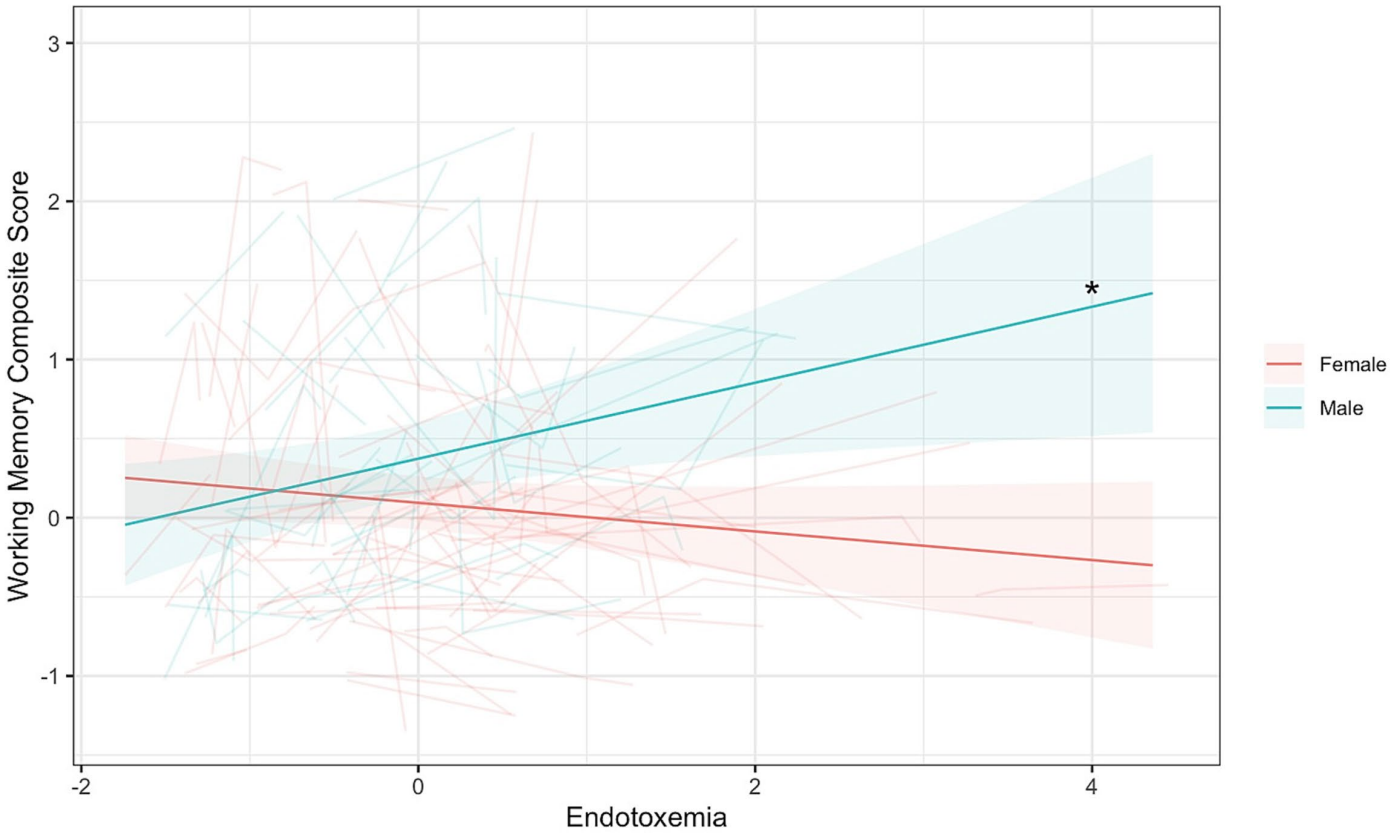
Endotoxemia and composite working memory were coupled across time differently for men and women (*B* = 0.33, [0.13, 0.53], *t*(172.01) = 3.26, *p* = 0.0013). Dark lines represent the fixed effects for men and women. Lighter lines represent each individual participant’s correlation. *Simple slopes, *p* < 0.05.

To reiterate, this coupling model seeks to explore if endotoxemia levels in a given wave correlate with cognitive function within the same wave. No significant coupling of endotoxemia with any of the cognitive tasks was observed as a main effect across time (Supplementary Tables S7, S8). However, a significant interaction between endotoxemia and gender was found in the multi-level coupling analysis for composite working memory (Endotoxemia × Gender: *B* = 0.33, [0.13, 0.53], *t*(172.01) = 3.26, *p* = 0.0013; Figure 2; Table 5).^3^ Simple slope analyses demonstrated a significant, positive association between endotoxemia and working memory performance for men (*B* = 0.24, *t*(197.72) = 2.50, *p* = 0.013; Figure 2), suggesting that higher levels of endotoxemia in a given wave correlated with better working memory performance in the same wave. A non-significant, negative association was evident for women (*B* = −0.09, *t*(109.20) = −1.55, *p* = 0.12, Figure 2), suggesting that endotoxemia and working memory were not correlated in a given wave. See Supplementary section for results from each individual task (Supplementary Tables S9, S10).

**Table 5:** Gender moderates coupling of endotoxemia with composite working memory.

	Composite working memory		
Predictors	Estimates	CI	*p*
(Intercept)	1.24	0.48–2.00	**0.002**
Time Linear	0.16	0.04–0.28	**0.010**
Time Quadratic	0.04	−0.06 to 0.14	0.438
LBP:CD14	−0.09	−0.21 to 0.02	0.122
Gender	0.28	0.02–0.53	**0.033**
Age	−0.02	−0.03 to −0.01	**<0.001**
Race	−0.15	−0.45 to 0.15	0.332
Ethnicity	−0.20	−0.54 to 0.13	0.234
Education	0.26	0.02–0.50	**0.035**
Wave 1 BMI	−0.01	−0.02 to 0.01	0.491
LBP:CD14 × Gender	0.33	0.13–0.53	**0.001**
Random effects			
σ^2^	0.22		
τ_00 Id_	0.45		
τ_11 Id.LBP_CD14_	0.00		
ρ_01 Id_	−1.00		
N _Id_	162		
Observations	330		
Marginal *R*^2^/Conditional *R*^2^	0.306/NA		

Bolded values indicate statistical significance, *p* < 0.05.

### 3.4 Follow-up analyses: menopause

We examined whether women’s menopausal status at baseline (pre-vs. post-menopausal women) influenced the relationship between gender, endotoxemia, and their interaction on working memory in both the prospective and coupling analyses described above. The previously described results for laboratory-assessed working memory were not altered by controlling for menopausal status (Supplementary Tables S11–S14).

One additional interactive effect was discovered in the prospective analyses, which explored whether baseline endotoxemia levels predicted changes in cognition across time: The association of endotoxemia with prospective changes in ambulatory spatial working memory significantly differed between pre-and post-menopausal women (Time_Linear_ × Pre vs. Post-Menopause × Endotoxemia: *B* = 0.055, [0.005, 0.105], *t*(93.13) = 2.12, *p* = 0.037; Supplementary Figure S7, Supplementary Table S12). For post-menopausal women, lower endotoxemia was associated with improved performance over time (i.e., practice effects) (*B* = −0.18, *t*(87.29), = −3.31, *p* = 0.001; Supplementary Figure S7), whereas post-menopausal women with higher endotoxemia did not exhibit this association (i.e., no practice effects on the spatial working memory task; *B* = −0.015, *t*(94.10) = −0.23, *p* = 0.82; Supplementary Figure S7). This association between endotoxemia levels and practice effects was not evident for (1) pre-menopausal women, who exhibited at least marginal improvement in spatial working memory across the spectrum of endotoxemia (*p*s < 0.08); or (2) for men, whose prospective performance was unrelated to endotoxemia levels in these analyses (*p*s > 0.30). It should be noted that this finding is exploratory and is based on a relatively small sample of post-menopausal women. Thus, this finding should be interpreted with caution and examined in larger samples specifically designed to examine the gut-brain axis and cognition in relation to menopause.

## 4 Discussion

This study explored endotoxemia as a potential contributing factor to differences in multiple aspects of cognitive functioning in healthy adults. It was hypothesized that higher levels of endotoxemia, indexed by the LBP:sCD14 ratio, would correlate with worsened performance on cognitive tasks, especially those related to attention and executive function (Madison et al., 2020; Monnig et al., 2017; Ahluwalia et al., 2014). This hypothesis was partly supported by prospective analyses, in which higher baseline endotoxemia predicted a lack of expected practice effects on a composite score of working memory. Those with lower endotoxemia improved across three measurements, each spaced approximately 9 months apart. Coupling analyses further revealed a gender difference in the correlation of endotoxemia with working memory. For men, higher levels of endotoxemia correlated with better working memory composite scores; for women, endotoxemia was not associated with working memory function. Put another way, at higher endotoxemia levels, men demonstrated better working memory function than women, with no evidence in improvement across time for either men or women; lower endotoxemia levels were associated with equivalent working memory performance for men and women, and similar improvements in working memory with practice (i.e., across 3 measurements in approximately 18 months). It is important to note that although we saw gender differences in the coupling model but not the prospective model, this does not inherently mean that there are not gender differences in how endotoxemia predicts working memory performance; we just did not detect them in this sample.

### 4.1 Endotoxemia may disrupt practice effects

The prospective analysis of endotoxemia revealed differences in change in working memory performance across time based on baseline levels of endotoxemia. Individuals with medium and lower levels of endotoxemia displayed working memory performance improvement across time, whereas those with higher levels of endotoxemia did not. As stated previously, the most evident reason for improvement in WM performance across the three timepoints is practice effects. Such effects are well-characterized in the literature that explores longitudinal cognitive testing, specifically as it relates to age-related cognitive change (Salthouse, 2010; Bartels et al., 2010). The longitudinal retest effects of working memory tasks are less well known, but work on executive function tasks and other tests of cognitive ability indicate that these practice effects can emerge even as far as 12 months to several years after the first administration of a test (Basso et al., 1999; Salthouse et al., 2004; Scharfen et al., 2018).

In a healthy, relatively young sample such as this one, practice effects may be a key sign of healthy cognitive functioning whereas a reduction in or absence of practice effects may be an early risk factor for cognitive decline. Prior literature has repeatedly found associations between reduced practice effects and worsened cognitive outcomes in clinical or preclinical samples. In individuals with MCI, the absence of practice effects has been shown to predict a worse prognosis for the condition (Duff et al., 2011). Subjects with preclinical AD also display impaired practice effects, with the magnitude of the practice effects present being inversely correlated with progression to disease (Hassenstab et al., 2015). Similar results have been seen in non-clinical, potentially at-risk individuals; those carrying the APOE4 genotype (associated with increased AD risk) displayed reduced practice effects on longitudinal cognitive measures compared to individuals without these alleles (Donix et al., 2012). Although we do not possess information about the cognitive outcomes of these participants in the long term, the prior literature suggests that the lack of practice effects seen for individuals with higher levels of endotoxemia at baseline may be indicative of an increased risk for negative cognitive outcomes later in life, including cognitive decline.

The main mechanism through which endotoxemia may contribute to a lack of practice effects is through inflammation. In prior research, elevated systemic inflammation induced via exercise or vaccination attenuated practice effects on a measure of processing speed and working memory (Paine et al., 2015). More broadly, elevated levels of endogenous inflammation (MCP-1, IL-6, CRP) have been associated with worse cognitive performance, especially executive function, in both cross-sectional and longitudinal study designs (Stenfors et al., 2017; Beydoun et al., 2018). The measure of endotoxemia utilized in this study is thought to estimate the degree of inflammatory response to endotoxins in the blood, most of which originate from the gut (Laugerette et al., 2014), but may also enter the bloodstream from the respiratory tract, other mucosal systems, and from infection (André et al., 2019). Hence, higher levels of this LBP:sCD14 ratio at baseline could indicate heightened inflammatory exposure over time, which may influence working memory performance, evident as reductions in practice effects. Other work has also suggested that gut dysbiosis (i.e., imbalance in the microbiome, of which a high LBP:sCD14 ratio could potentially be an indicator) can lead to increases in anhedonia and amotivation, which may be another potential explanation for the lack of expected improvements in working memory performance across time for individuals with higher LBP:sCD14 seen here (Safadi et al., 2022). Future work that combines microbiome measurement with endotoxemia measures will be able to examine this possibility more specifically.

### 4.2 Gender differences in the relationship between endotoxemia and cognition

The observed gender differences in the relationship between endotoxemia and working memory in the coupling model were surprising, but there is some basis for this in prior literature. Past analyses of the ESCAPE dataset found that endotoxemia produced a stronger, positive correlation between *ex vivo* stimulated inflammation and depression symptomatology in men but not in women, who showed weak negative correlations between inflammation and depressive symptoms at higher endotoxemia levels (Knight et al., 2020). Previous research in rats has found that female rats desensitize to repeated LPS administrations faster than male rats (Engeland et al., 2006; Cloutier et al., 2018). This suggests that women may be less likely to show strong reactions, positive or negative, to endotoxemia, even in the face of a greater proinflammatory predisposition measured by heightened LBP:sCD14 ratios.

Prior literature has revealed that endotoxins may help prime the brain to respond in situations requiring attentional control in men. Low-dose administration of endotoxins (LPS) is associated with changes in alpha and beta neural activity indicative of increased alertness (van den Boogaard et al., 2010). Additionally, high-dose LPS administration has been shown to decrease reaction time on the N-Back test (Grigoleit et al., 2011). Circulating levels of LPS have also been connected to improved performance on measures of executive function in men, though this finding is limited by small sample size and participant comorbidities, including HIV and alcohol use disorder (Monnig et al., 2017). Here, we provide evidence that the LBP:sCD14 ratio, which is a simultaneous index of naturally occurring exposure to endotoxins and the immune system’s predisposition to mount an inflammatory response, correlates positively with working memory function in men. Collectively, these findings suggest a pathway in which endotoxins may act as an alerting signal to the brain, ultimately priming working memory in the short term. To reiterate, comparison between LPS administration and endotoxemia measurement is difficult due to measurement difficulties with LPS (Novitsky, 1998) and the varying stabilities of these molecules (Yao et al., 2016; Turgunov et al., 2024). We hypothesize that the pathway allowing for this “alerting signal” can be situated within the acute stress response model. Glutamatergic transmission in the prefrontal cortex (PFC) increases during acute stress—increases that can have beneficial effects on working memory (Yuen et al., 2009). This increased activation of the PFC is thought to arise from dual activation of the corticosteroid response (HPA axis) and sympathetic nervous system (SNS) in response to stress (Yuen et al., 2009; Dalise et al., 2020). In rats, circulating adrenal corticosteroids released as part of the stress response increase gut permeability (Meddings and Swain, 2000). Other work has hypothesized that the elevated proinflammatory response to acute stress arises primarily from a stress-induced increase in epithelial permeability in the gut (de Punder and Pruimboom, 2015). This pro-inflammatory response interacts with the sympathetic nervous system, which can promote or regulate the response in a context-dependent manner (Ribeiro-da-Silva et al., 2018). The putative interaction between the SNS, the HPA axis, and endotoxemia could allow for stress-induced changes in cognition for men. Although this theory has yet to be substantiated, it provides a framework for situating endotoxemia as a component of the acute stress response, which is already known to produce acute working memory boosts.

Prior work that has explored short term fluctuations in LBP and sCD14 has mixed results. Some have found fluctuations in LBP (Laugerette et al., 2020; Zaman and Zaman, 2015; Pei et al., 2018), sCD14 (Hall et al., 2024), or their ratio (Pei et al., 2018), while others have found no evidence of short term changes in these measures (e.g., postprandially; Camargo et al., 2019; Madison et al., 2020; López-Moreno et al., 2017). There is also preliminary evidence that acute stress (such as an administration of CRH or intense exercise) can transiently affect levels of LBP and/or sCD14 in humans (Mogilevski et al., 2024; Fitzpatrick et al., 2024; Aune et al., 2021), though the strength and direction of these relationships requires further study. Examining under what circumstances short-term changes in gut permeability occur and how these fluctuations correlate with working memory could help clarify the association found in this report.

We hypothesize that acute stress may help explain the positive association seen between endotoxemia and working memory in men, but it is unclear exactly what this acute stressor may be. Previous research has found that both blood draws and cognitive testing can lead to an acute stress response, but the degree of such a response is subject to many individual differences (Weckesser et al., 2014; Seeman et al., 2001; Neupert et al., 2006). It seems plausible that alterations in endotoxemia would occur on a similar timescale to HPA axis and SNS responses, but no work has evaluated such a claim. Although women may be subject to the same stress-induced increase in endotoxemia during a blood draw, cognitive testing, or other life stressors, they may not experience the same cognitive outcomes due to the desensitization of the immune response to repeated exposures to endotoxins discussed previously.

Importantly, our hypothesizing focuses on relatively short-term effects. It is unlikely that heightened markers of endotoxemia are beneficial for men in the long term. Both the prospective model detailed in this project and numerous other studies have found evidence that elevated LBP, sCD14, and their ratio are negatively associated with white matter connectivity and performance on attention tasks (Moreno-Navarrete et al., 2017; Madison et al., 2020). Elevated fasting levels of LPS are also positively associated with Alzheimer’s Disease and related dementias (André et al., 2019). It remains unclear at what point elevated levels of these biomarkers can become problematic for men and what the timescale for this transition from potentially beneficial short-term elevations to more chronic, harmful inflammatory activity might be.

### 4.3 Discrepancies in ambulatory vs. lab cognition

The proposed function of elevated endotoxemia as a component of the acute stress response could explain why we do not see an association between LBP:sCD14 and working memory in the ambulatory cognitive tasks (with the exception of the exploratory findings relating menopause and spatial working memory). One of the proposed benefits of ambulatory assessment for cognitive measures is that it may reduce some of the stress associated with in-laboratory cognitive assessments (Crawford et al., 2022). If endotoxemia is associated with improved working memory outcomes for men via the stress response, we may not see such effects in ambulatory tasks which are theoretically less stressful for participants.

Although this stress explanation appears to be plausible, there are other factors that may explain why an association between endotoxemia and working memory was observed in the laboratory-based tasks, but not in the ambulatory tasks. First, the laboratory and ambulatory working memory tasks assess somewhat different components of working memory (verbal/numeric working memory in lab, verbal/spatial working memory in ambulatory), although previous research with the ESCAPE dataset has found correlations between ambulatory and laboratory cognitive data (Sliwinski et al., 2018). There are also important methodological differences between ambulatory and laboratory cognitive tasks. It is thought that ambulatory data provide better insight into people’s *average* cognitive performance, whereas laboratory tasks are designed to reveal people’s *maximum* performance (Sliwinski et al., 2018). Perhaps endotoxemia in healthy adult men is only associated with working memory performance at “extremes” (i.e., in the laboratory), while typical performance (i.e., ambulatory) remains unchanged. Prior research has indicated the need for more advanced statistical analyses to address retest effects in ambulatory cognitive data, such as Bayesian double exponential learning curve modeling (Oravecz et al., 2022). Such models were not utilized in this study, and this may limit our ability to understand the relationship between ambulatory working memory and endotoxemia. Follow-up work is necessary to clarify the differences in the association between ecologically-vs. laboratory-assessed working memory and endotoxemia.

### 4.4 Limitations and future directions

One significant limitation is that the present sample was limited to physically healthy individuals who had not been diagnosed with a major chronic health condition, including autoimmune conditions. Although this decision was valuable in that it enabled us to explore typical immune responses (Knight et al., 2020) and thus characterize how endotoxemia may affect healthy individuals, it significantly limits generalizability, as around 60% of Americans live with a chronic health condition of some kind (Centers for Disease Control and Prevention, 2023). Similarly, the ESCAPE dataset involved a sample that was generally cognitively healthy at baseline, which allowed us to explore the role of endotoxemia in cognition in non-diseased states. However, it would also be valuable for similar research to be conducted in a sample with neurodegenerative conditions given that prior research has found connections between endotoxemia and neurodegeneration (Brown, 2019; André et al., 2019; Mizobuchi and Soma, 2021). Along with observing this relationship in clinical populations, it would be beneficial to determine at what threshold chronically elevated levels of these biomarkers become problematic and can contribute to neurodegeneration or other clinical conditions.

Another limitation is the lack of measures of attention. Prior literature found associations between endotoxemia, attention, and executive function (Madison et al., 2020; Monnig et al., 2017). Working memory and attention are closely related; attention functions as a “gatekeeper” to working memory and is integral for maintaining representations in active use (Awh et al., 2006). Further, most measures of working memory have a strong positive relationship with measures of attentional control (Panichello and Buschman, 2021). Although attention, executive function, and working memory are undoubtedly related, they are not the same. Because there are no measures of attention in the ESCAPE dataset, it is difficult to meaningfully compare these findings to prior research on the topic. It would be valuable for future work to include measures of attention, executive function, and working memory within the same study to better characterize how endotoxemia may differentially relate to these constructs.

An additional limitation is the timescale on which study measures were collected. Measures of laboratory cognition were taken around 2 weeks prior to the blood sample that provided levels of LBP and sCD14. Prior work has suggested that LBP has moderate test–retest reliability within a span of 1 week to several months, with the reliability improving with closer time intervals (Citronberg et al., 2016). The ICCs seen in this study for LBP and sCD14 also demonstrate within-participant reliability for LBP:sCD14 across longer periods of time (i.e., 9 to 18 months). This suggests that LBP:sCD14 is a relatively stable measure; it can be assumed that levels of these two biomarkers do not fluctuate significantly in the two-week span between cognitive testing and plasma collection. However, this study design limits our understanding of what role acute stress (such as the blood draw and cognitive testing) has on the relationship between LBP:sCD14 and working memory. Understanding these connections is likely critical to explaining the results seen here. Future work would benefit from replicating this study design with (i) a shorter interval between cognitive testing and the collection of blood plasma; (ii) a better understanding the time course of changes in endotoxemia measures due to stress; and (iii) potentially including exposure to an acute stressor to further substantiate the results seen here and better characterize the connection between endotoxemia and working memory.

It could also be beneficial to explore this relationship in a larger sample to better characterized the effects gender, race, BMI, education, or other demographic factors. This study was observational in design and recruited to represent the geographic area from which participants were drawn. This makes these findings generalizable to a diverse population and is a strength of this design. Our power simulations suggest the sample was well-powered to detect an effect of gender despite the unequal sample sizes. However, the findings in this report with regards to gender differences in the relationship between endotoxemia and working memory could be improved by recruiting larger samples of men and women, which would improve our confidence in the magnitude and direction of the gender moderation findings.

Another important avenue to examine in future endotoxemia research is the contribution to, and interactions with, inflammation and the gut microbiome.^4^ In animal work, differences in the bacterial strain and structure of LPS administered has been shown to contribute to the outcome of the immune response, with some strains actually conferring a metabolic benefit in rats (Anhê et al., 2021). Perhaps the strains of bacteria translocating from the gut into the blood in human endotoxemia matter as well. Exploring the connections between gut microbiota, endotoxemia levels, and the inflammatory response to endotoxemia is an important next step that may provide a better understanding of how these processes contribute to cognitive health.

## 5 Conclusion

We found evidence of both improved and reduced cognitive functioning associated with endotoxemia. In prospective analyses, higher levels of endotoxemia at baseline were correlated with a lack of expected improvement in working memory performance (i.e., practice effects) across about 18 months of measurement. However, in the coupling analysis, men with higher levels of endotoxemia performed better on working memory tasks, potentially through the acute stress response mechanism. Women displayed no such association, which may result from females’ propensity toward a greater adaptive immune response and more rapid desensitization to repeated exposure to endotoxins (Engeland et al., 2006; Vázquez-Martínez et al., 2018). It is also possible that gender is just one of many biopsychosocial individual differences that may modulate the extent to which endotoxemia associates with working memory or other cognition functioning. Future work should continue to explore gender, as well as age, physical and cognitive health status, and gut microbiota characteristics to best characterize the connections between endotoxemia and cognitive health outcomes.

Ultimately, this basic science provides novel insights into the longitudinal associations between the gut and immune system that may impact cognitive health in diverse populations. Although the findings of this project are preliminary, they provide evidence of (1) prospective effects of endotoxemia on changes in working memory and (2) gender-based differences in how endotoxemia correlates with working memory. More work is needed to establish how endotoxemia may facilitate communication between the gut, immune system, and brain, and what the ramifications of this integrated system might be for cognitive aging.

## Data Availability

The raw data supporting the conclusions of this article will be made available by the authors, without undue reservation.

## Footnotes

^1^A fourth ESCAPE wave was started but was not completed, and endotoxemia biomarkers are not available from this wave.

^2^This model converged with a singular fit, suggesting the variance of the linear effects of time are (close to) zero. A model with random slopes removed was run, which fully replicated the results reported here (B = −0.100, [−0.195, −0.006], t(195.92) = −2.078, *p* = 0.039).

^3^As with the prospective model, the coupling model also demonstrated evidence of a singularity (i.e., low variance explained at the individual level). A model with random slopes for endotoxemia replicated the model reported (B = −0.318, [−0.518, −0.118], t (306.63) = −3.116, *p* = 0.002). We retained the model with singular-fit specifically because of the importance of estimating the individual slopes for each participant in the coupling model.

^4^The ESCAPE study has measurements of inflammation (Scott et al., 2015). However, analyses producing a comprehensive, multilevel structural equation model were deemed overly burdensome for the present report, which aimed to examine initial associations. Exploring the connections between LBP:sCD14, inflammation, and cognitive functioning is a next step that can be accomplished with this sample.

## References

[ref1] Ahluwalia V. Wade J. B. Heuman D. M. Hammeke T. A. Sanyal A. J. Sterling R. K.. (2014). Enhancement of functional connectivity, working memory and inhibitory control on multi-modal brain MR imaging with Rifaximin in cirrhosis: implications for the gut-liver-brain axis. Metab. Brain Dis. 29, 1017–1025. doi: 10.1007/s11011-014-9507-6, PMID: 24590688 PMC4155029

[ref2] André P. Laugerette F. Féart C. (2019). Metabolic Endotoxemia: a potential underlying mechanism of the relationship between dietary fat intake and risk for cognitive impairments in humans? Nutrients 11:8. doi: 10.3390/nu11081887, PMID: 31412673 PMC6722750

[ref3] Anhê F. F. Barra N. G. Cavallari J. F. Henriksbo B. D. Schertzer J. D. (2021). Metabolic endotoxemia is dictated by the type of lipopolysaccharide. Cell Rep. 36:109691. doi: 10.1016/j.celrep.2021.109691, PMID: 34525353

[ref4] Aune S. K. Cwikiel J. Flaa A. Arnesen H. Solheim S. Awoyemi A.. (2021). Gut leakage markers in response to strenuous exercise in patients with suspected coronary artery disease. Cells 10:2193. doi: 10.3390/cells10092193, PMID: 34571843 PMC8466709

[ref5] Awh E. Vogel E. K. Oh S. H. (2006). Interactions between attention and working memory. Neuroscience 139, 201–208. doi: 10.1016/j.neuroscience.2005.08.023 16324792

[ref6] Bartels C. Wegrzyn M. Wiedl A. Ackermann V. Ehrenreich H. (2010). Practice effects in healthy adults: a longitudinal study on frequent repetitive cognitive testing. BMC Neurosci. 11:118. doi: 10.1186/1471-2202-11-118, PMID: 20846444 PMC2955045

[ref7] Basso M. R. Bornstein R. A. Lang J. M. (1999). Practice effects on commonly used measures of executive function across twelve months. Clin. Neuropsychol. 13, 283–292. doi: 10.1076/clin.13.3.283.1743, PMID: 10726600

[ref8] Bates D. Mächler M. Bolker B. Walker S. (2015). Fitting linear mixed-effects models using lme 4. J. Stat. Softw. 67:1. doi: 10.18637/jss.v067.i01

[ref9] Beydoun M. A. Dore G. A. Canas J. A. Liang H. Beydoun H. A. Evans M. K.. (2018). Systemic inflammation is associated with longitudinal changes in cognitive performance among urban adults. Front. Aging Neurosci. 10:313. doi: 10.3389/fnagi.2018.00313, PMID: 30356710 PMC6189312

[ref10] Binder L. M. Villanueva M. R. Howieson D. Moore R. T. (1993). The rey avlt recognition memory task measures motivational impairment after mild head trauma. Arch. Clin. Neuropsychol. 8, 137–147. doi: 10.1093/arclin/8.2.137 14589671

[ref11] Brown G. C. (2019). The endotoxin hypothesis of neurodegeneration. J. Neuroinflammation 16:180. doi: 10.1186/s12974-019-1564-7, PMID: 31519175 PMC6744684

[ref12] Camargo A. Jimenez-Lucena R. Alcala-Diaz J. F. Rangel-Zuñiga O. A. Garcia-Carpintero S. Lopez-Moreno J.. (2019). Postprandial endotoxemia may influence the development of type 2 diabetes mellitus: from the CORDIOPREV study. Clin. Nutr. 38, 529–538. doi: 10.1016/j.clnu.2018.03.016 29685478

[ref13] Carabotti M. Scirocco A. Maselli M. A. Severi C. (2015). The gut-brain axis: interactions between enteric microbiota, central and enteric nervous systems. Ann. Gastroenterol. 282, 203–209. PMC4367209 25830558

[ref14] Case R. Kurland D. M. Goldberg J. (1982). Operational efficiency and the growth of short-term memory span. J. Exp. Child Psychol. 33, 386–404. doi: 10.1016/0022-0965(82)90054-6

[ref15] Centers for Disease Control and Prevention (2023). Chronic disease. Available at: https://www.cdc.gov/chronicdisease/index.htm#:~:text=Six%20in%20ten%20Americans%20live,stroke%2C%20cancer%2C%20or%20diabetes (Accessed October 5, 2023).

[ref16] Citronberg J. S. Wilkens L. R. Lim U. Hullar M. A. White E. Newcomb P. A.. (2016). Reliability of plasma lipopolysaccharide-binding protein (LBP) from repeated measures in healthy adults. Cancer Causes Control 27, 1163–1166. doi: 10.1007/s10552-016-0783-9, PMID: 27392432 PMC5068910

[ref17] Cloutier C. J. Kavaliers M. Ossenkopp K. P. (2018). Lipopolysaccharide (LPS) induced sickness in adolescent female rats alters the acute-phase response and lithium chloride (LiCl)- induced impairment of conditioned place avoidance/aversion learning, following a homotypic LPS challenge in adulthood. Behav. Brain Res. 351, 121–130. doi: 10.1016/j.bbr.2018.05.033, PMID: 29885379

[ref18] Colom R. Abad F. J. Rebollo I. Shih P. C. (2005). Memory span and general intelligence: a latent-variable approach. Intelligence 33, 623–642. doi: 10.1016/j.intell.2005.05.006

[ref19] Conway A. R. A. Kane M. J. Bunting M. F. Hambrick D. Z. Wilhelm O. Engle R. W. (2005). Working memory span tasks: a review and a user’s guide. Psychon. B Rev. 12:769. doi: 10.3758/BF03196772 16523997

[ref20] Cooke Bailey J. N. Bush W. S. Crawford D. C. (2020). The importance of diversity in precision medicine research. Front. Genet. 11:875. doi: 10.3389/fgene.2020.00875, PMID: 33005167 PMC7479241

[ref21] Crawford J. L. English T. Braver T. S. (2022). Incorporating ecological momentary assessment into multimethod investigations of cognitive aging: promise and practical considerations. Psychol. Aging 37, 84–96. doi: 10.1037/pag0000646, PMID: 35113616 PMC8860503

[ref22] Dalise A. M. Prestano R. Fasano R. Gambardella A. Barbieri M. Rizzo M. R. (2020). Autonomic nervous system and cognitive impairment in older patients: evidence from long-term heart rate variability in real-life setting. Front. Aging Neurosci. 12:40. doi: 10.3389/fnagi.2020.00040 32218729 PMC7079686

[ref23] Darnall B. D. Suarez E. C. (2009). Sex and gender in psychoneuroimmunology research: past, present and future. Brain Behav. Immun. 23, 595–604. doi: 10.1016/j.bbi.2009.02.019, PMID: 19272440 PMC2740642

[ref24] de Punder K. Pruimboom L. (2015). Stress induces endotoxemia and low-grade inflammation by increasing barrier permeability. Front. Immunol. 6:223. doi: 10.3389/fimmu.2015.00223, PMID: 26029209 PMC4432792

[ref25] Donix M. Ercoli L. M. Siddarth P. Brown J. A. Martin-Harris L. Burggren A. C.. (2012). Influence of Alzheimer disease family history and genetic risk on cognitive performance in healthy middle-aged and older people. Am. J. Geriatr. Psychiatry 20, 565–573. doi: 10.1097/JGP.0b013e3182107e6a, PMID: 21849821 PMC3816758

[ref26] Donoso F. Cryan J. F. Olavarría-Ramírez L. Nolan Y. M. Clarke G. (2023). Inflammation, lifestyle factors, and the microbiome-gut-brain axis: relevance to depression and antidepressant action. Clin. Pharmacol. Ther. 113, 246–259. doi: 10.1002/cpt.2581, PMID: 35278334 PMC10084001

[ref27] Duff K. Lyketsos C. G. Beglinger L. J. Chelune G. Moser D. J. Arndt S.. (2011). Practice effects predict cognitive outcome in amnestic mild cognitive impairment. Am. J. Geriatr. Psychiatry 19, 932–939. doi: 10.1097/JGP.0b013e318209dd3a, PMID: 22024617 PMC3202689

[ref28] El-Lakany M. A. Fouda M. A. El-Gowelli H. M. El-Gowilly S. M. El-Mas M. M. (2018). Gonadal hormone receptors underlie the resistance of female rats to inflammatory and cardiovascular complications of endotoxemia. Eur. J. Pharmacol. 823, 41–48. doi: 10.1016/j.ejphar.2018.01.051 29382531

[ref29] Engeland C. G. Kavaliers M. Ossenkopp K.-P. (2006). Influence of the estrous cycle on tolerance development to LPS-induced sickness behaviors in rats. Psychoneuroendocrinology 31, 510–525. doi: 10.1016/j.psyneuen.2005.11.007 16413135

[ref30] Fitzpatrick J. A. Gibson P. R. Taylor K. M. Halmos E. P. (2024). The effect of dietary emulsifiers and thickeners on intestinal barrier function and its response to acute stress in healthy adult humans: a randomised controlled feeding study. Aliment. Pharmacol. Ther. 60, 863–875. doi: 10.1111/apt.18172, PMID: 39072856

[ref31] Gonzalez-Quintela A. Alonso M. Campos J. Vizcaino L. Loidi L. Gude F. (2013). Determinants of serum concentrations of lipopolysaccharide-binding protein (LBP) in the adult population: the role of obesity. PLoS One 8:e54600. doi: 10.1371/journal.pone.0054600, PMID: 23349936 PMC3551812

[ref32] Grigoleit J. S. Kullmann J. S. Wolf O. T. Hammes F. Wegner A. Jablonowski S.. (2011). Ose-dependent effects of endotoxin on neurobehavioral functions in humans. PLoS One 6:e28330. doi: 10.1371/journal.pone.0028330, PMID: 22164271 PMC3229570

[ref33] Hall W. L. Alkoblan A. Gibson P. S. D'Annibale M. Coekaerts A. Bauer M.. (2024). Postprandial lipid and vascular responses following consumption of a commercially-relevant interesterified palmitic acid-rich spread in comparison to functionally-equivalent non-interesterified spread and spreadable butter: a randomised controlled trial in healthy adults. Food Funct. 15, 2733–2750. doi: 10.1039/d3fo05324e, PMID: 38380649 PMC10911404

[ref34] Han B. Hejun L. Shan X. Sun Q. Fan W. (2016). PT560. A comparative study of serum tau protein and Aß levels on intestinal endotoxemia among Alzheimer’s disease rats and in Chinese sample of Alzheimer’s disease patients and healthy controls. Int. J. Neuropsychopharmacol. 19:7. doi: 10.1093/ijnp/pyw044.560

[ref1002] Hassenstab J. Ruvolo D. Jasielec M. Xiong C. Grant E. Morris J. C. (2015). Absence of practice effects in preclinical Alzheimer’s disease. Neuropsychology. 29:6. doi: 10.1037/neu0000208, PMID: 26011114 PMC4640964

[ref38] Kiecolt-Glaser J. K. Wilson S. J. Bailey M. L. Andridge R. Peng J. Jaremka L. M.. (2018). Marital distress, depression, and a leaky gut: translocation of bacterial endotoxin as a pathway to inflammation. Psychoneuroendocrinology 98, 52–60. doi: 10.1016/j.psyneuen.2018.08.007, PMID: 30098513 PMC6260591

[ref39] Kitchens R. L. Thompson P. A. (2005). Modulatory effects of sCD14 and LBP on LPS-host cell interactions. J. Endotoxin Res. 11:4. doi: 10.1177/0968051905011004070 16176659

[ref40] Knight E. L. Majd M. Graham-Engeland J. E. Smyth J. M. Sliwinski M. J. Engeland C. G. (2020). Gender differences in the link between depressive symptoms and ex vivo inflammatory responses are associated with markers of endotoxemia. Brain Behav. Immun. Health 2:100013. doi: 10.1016/j.bbih.2019.100013, PMID: 34258602 PMC8274590

[ref41] Kuznetsova A. Brockhoff P. B. Christensen R. H. B. (2017). lmerTest package: tests in linear mixed effects models. J. Stat. Softw. 82:13. doi: 10.18637/jss.v082.i13

[ref42] Laugerette F. Alligier M. Bastard J. Drai J. Chanséaume E. Lambert-Porcheron S.. (2014). Overfeeding increases postprandial endotoxemia in men: inflammatory outcome may depend on LPS transporters LBP and sCD14. Mol. Nutr. Food Res. 58, 1513–1518. doi: 10.1002/mnfr.201400044, PMID: 24687809

[ref43] Laugerette F. Vors C. Alligier M. Pineau G. Drai J. Knibbe C.. (2020). Postprandial endotoxin transporters LBP and sCD14 differ in obese vs. overweight and normal weight men during fat-rich meal digestion. Nutrients 12:6.1820. doi: 10.3390/nu12061820, PMID: 32570947 PMC7353369

[ref45] Liu P. Gao M. Liu Z. Zhang Y. Tu H. Lei L.. (2022). Gut microbiome composition linked to inflammatory factors and cognitive functions in first-episode, drug-naive major depressive disorder patients. Front. Neurosci. 15:e800764. doi: 10.3389/fnins.2021.800764, PMID: 35153660 PMC8831735

[ref46] Looijer-van Langen M. Hotte N. Dieleman L. A. Albert E. Mulder C. Madsen K. L. (2011). Estrogen receptor-β signaling modulates epithelial barrier function. Am. J. Physiol. Gastrointest. Liver Physiol. 300, G621–G626. doi: 10.1152/ajpgi.00274.2010, PMID: 21252046

[ref47] López-Moreno J. García-Carpintero S. Jimenez-Lucena R. Haro C. Rangel-Zúñiga O. A. Blanco-Rojo R.. (2017). Effect of dietary lipids on endotoxemia influences postprandial inflammatory response. J. Agric. Food Chem. 65, 7756–7763. doi: 10.1021/acs.jafc.7b01909, PMID: 28793772

[ref48] Madison A. A. Belury M. A. Andridge R. Shrout M. R. Renna M. E. Malarkey W. B.. (2020). Afternoon distraction: a high-saturated-fat meal and endotoxemia impact postmeal attention in a randomized crossover trial. Am. J. Clin. Nutr. 111, 1150–1158. doi: 10.1093/ajcn/nqaa085 32393980 PMC7266694

[ref49] Marceau K. Ruttle P. L. Shirtcliff E. A. Hastings P. D. Klimes-Dougan B. Zahn-Waxler C. (2015). Within-person coupling of changes in cortisol, testosterone, and DHEA across the day in adolescents. Dev. Psychobiol. 57, 654–669. doi: 10.1002/dev.21173 24166536 PMC4000581

[ref50] Marsland A. L. Gianaros P. J. Kuan D. C. H. Sheu L. K. Krajina K. Manuck S. B. (2015). Brain morphology links systemic inflammation to cognitive function in midlife adults. Brain Behav. Immun. 48, 195–204. doi: 10.1016/j.bbi.2015.03.015, PMID: 25882911 PMC4508197

[ref52] McAfoose J. Baune B. T. (2009). Evidence for a cytokine model of cognitive function. Neurosci. Biobehav. Rev. 33, 355–366. doi: 10.1016/j.neubiorev.2008.10.005 18996146

[ref53] Meddings J. B. Swain M. G. (2000). Environmental stress-induced gastrointestinal permeability is mediated by endogenous glucocorticoids in the rat. Gastroenterology 119, 1019–1028. doi: 10.1053/gast.2000.18152, PMID: 11040188

[ref54] Misiak B. Beszłej J. A. Kotowicz K. Szewczuk-Bogusławska M. Samochowiec J. Kucharska-Mazur J.. (2018). Cytokine alterations and cognitive impairment in major depressive disorder: from putative mechanisms to novel treatment targets. Prog. Neuro Psychopharmacol. Biol. Psychiatry 80, 177–188. doi: 10.1016/j.pnpbp.2017.04.021, PMID: 28433456

[ref55] Mizobuchi H. Soma G. I. (2021). Low-dose lipopolysaccharide as an immune regulator for homeostasis maintenance in the central nervous system through transformation to neuroprotective microglia. Neural Regen. Res. 16, 1928–1934. doi: 10.4103/1673-5374.308067, PMID: 33642362 PMC8343302

[ref56] Mogilevski T. Rosella S. Nguyen A. Fitzpatrick J. Parker F. Halmos E. P.. (2024). Characterisation of biomarkers of intestinal barrier function in response to a high fat/high carbohydrate meal and corticotropin releasing hormone. PLoS One 19:e0294918. doi: 10.1371/journal.pone.0294918, PMID: 38408050 PMC10896497

[ref57] Monnig M. Kahler C. Cioe P. Monti P. Mayer K. Pantalone D.. (2017). Markers of microbial translocation and immune activation predict cognitive processing speed in heavy-drinking men living with HIV. Microorganisms 5:4. doi: 10.3390/microorganisms5040064, PMID: 28934108 PMC5748573

[ref58] Moreno-Navarrete J. M. Blasco G. Puig J. Biarnés C. Rivero M. Gich J.. (2017). Neuroinflammation in obesity: circulating lipopolysaccharide-binding protein associates with brain structure and cognitive performance. Int. J. Obes. 41, 1627–1635. doi: 10.1038/ijo.2017.162, PMID: 28684860

[ref59] Neupert S. D. Miller L. M. S. Lachman M. E. (2006). Physiological reactivity to cognitive stressors: variations by age and socioeconomic status. Int. J. Aging Hum. Dev. 62, 221–235. doi: 10.2190/17DU-21AA-5HUK-7UFG 16625938

[ref60] Novitsky T. J. (1998). Limitations of the Limulus amebocyte lysate test in demonstrating circulating lipopolysaccharides. Ann. N. Y. Acad. Sci. 851, 416–421. doi: 10.1111/j.1749-6632.1998.tb09018.x 9668634

[ref61] Odenwald M. A. Turner J. R. (2013). Intestinal permeability defects: is it time to treat? Clin. Gastroenterol. Hepatol. 11, 1075–1083. doi: 10.1016/j.cgh.2013.07.001, PMID: 23851019 PMC3758766

[ref62] Oravecz Z. Harrington K. D. Hakun J. G. Katz M. J. Wang C. Zhaoyang R.. (2022). Accounting for retest effects in cognitive testing with the Bayesian double exponential model via intensive measurement burst designs. Front. Aging Neurosci. 14:897343. doi: 10.3389/fnagi.2022.897343 36225891 PMC9549774

[ref63] Paine N. J. Bosch J. A. Ring C. Drayson M. T. Veldhuijzen van Zanten J. J. (2015). Induced mild systemic inflammation is associated with impaired ability to improve cognitive task performance by practice. Psychophysiology 52, 333–341. doi: 10.1111/psyp.12360, PMID: 25366393

[ref64] Pajkrt D. Doran J. E. Koster F. Lerch P. G. Arnet B. Van Der Poll T.. (1996). Anti-inflammatory effects of reconstituted high-density lipoprotein during human endotoxemia. J. Exp. Med. 184, 1601–1608. doi: 10.1084/jem.184.5.1601, PMID: 8920850 PMC2192853

[ref66] Panichello M. F. Buschman T. J. (2021). Shared mechanisms underlie the control of working memory and attention. Nature 592, 601–605. doi: 10.1038/s41586-021-03390-w, PMID: 33790467 PMC8223505

[ref67] Pei R. DiMarco D. M. Putt K. K. Martin D. A. Chitchumroonchokchai C. Bruno R. S.. (2018). Premeal low-fat yogurt consumption reduces postprandial inflammation and markers of endotoxin exposure in healthy premenopausal women in a randomized controlled trial. J. Nutr. 148, 910–916. doi: 10.1093/jn/nxy046, PMID: 29767743 PMC5991203

[ref68] Pluck G. Ruales-Chieruzzi C. B. Paucar-Guerra E. J. Andrade-Guimaraes M. V. Trueba A. F. (2016). Separate contributions of general intelligence and right prefrontal neurocognitive functions to academic achievement at university level. Trends Neurosci. Educ. 5, 178–185. doi: 10.1016/j.tine.2016.07.002

[ref69] Pollack M. Ohl C. A. Golenbock D. T. Di Padova F. Wahl L. M. Koles N. L.. (1997). Dual effects of LPS antibodies on cellular uptake of LPS and LPS-induced proinflammatory functions. J. Immunol. 159, 3519–3530. doi: 10.4049/jimmunol.159.7.3519 9317151

[ref70] Polo A. J. Makol B. A. Castro A. S. Colón-Quintana N. Wagstaff A. E. Guo S. (2019). Diversity in randomized clinical trials of depression: a 36-year review. Clin. Psychol. Rev. 67:22. doi: 10.1016/j.cpr.2018.09.004 30292439

[ref71] Preacher K. J. Curran P. J. Bauer D. J. (2006). Computational tools for probing interactions in multiple linear regression, multilevel modeling, and latent curve analysis. J. Educ. Behav. Stat. 31, 437–448. doi: 10.3102/10769986031004437

[ref72] Proctor C. Thiennimitr P. Chattipakorn N. Chattipakorn S. C. (2017). Diet, gut microbiota and cognition. Metab. Brain Dis. 32, 1–17. doi: 10.1007/s11011-016-9917-8 27709426

[ref73] R Core Team (2023). R: A language and environment for statistical computing. Vienna, Austria: R Foundation for Statistical Computing.

[ref75] Rea K. Dinan T. G. Cryan J. F. (2016). The microbiome: a key regulator of stress and neuroinflammation. Neurobiol. Stress. 4, 23–33. doi: 10.1016/j.ynstr.2016.03.001, PMID: 27981187 PMC5146205

[ref76] Ribeiro-da-Silva M. Vasconcelos D. M. Alencastre I. S. Oliveira M. J. Linhares D. Neves N.. (2018). Interplay between sympathetic nervous system and inflammation in aseptic loosening of hip joint replacement. Sci. Rep. 8:16044. doi: 10.1038/s41598-018-33360-8, PMID: 30375409 PMC6207762

[ref77] Safadi J. M. Quinton A. M. Lennox B. R. Burnet P. W. Minichino A. (2022). Gut dysbiosis in severe mental illness and chronic fatigue: a novel trans-diagnostic construct a systematic review and meta-analysis. Mol. Psychiatry 27, 141–153. doi: 10.1038/s41380-021-01032-1, PMID: 33558650 PMC8960409

[ref78] Salthouse T. A. (2010). Influence of age on practice effects in longitudinal neurocognitive change. Neuropsychology 24, 563–572. doi: 10.1037/a0019026, PMID: 20804244 PMC2933088

[ref79] Salthouse T. A. Schroeder D. H. Ferrer E. (2004). Estimating retest effects in longitudinal assessments of cognitive functioning in adults between 18 and 60 years of age. Dev. Psychol. 40, 813–822. doi: 10.1037/0012-1649.40.5.813, PMID: 15355168

[ref80] Scharfen J. Jansen K. Holling H. (2018). Retest effects in working memory capacity tests: a meta-analysis. Psychon. Bull. Rev. 25, 2175–2199. doi: 10.3758/s13423-018-1461-6, PMID: 29907925

[ref81] Scott S. B. Graham-Engeland J. E. Engeland C. G. Smyth J. M. Almeida D. M. Katz M. J.. (2015). The effects of stress on cognitive aging, physiology and emotion (ESCAPE) project. BMC Psychiatry 15:146. doi: 10.1186/s12888-015-0497-7, PMID: 26138700 PMC4490700

[ref82] Seeman T. E. Singer B. Wilkinson C. W. McEwen B. (2001). Gender differences in age-related changes in HPA axis reactivity. Psychoneuroendocrinology 26, 225–240. doi: 10.1016/s0306-4530(00)00043-3 11166486

[ref84] Sliwinski M. J. Mogle J. A. Hyun J. Munoz E. Smyth J. M. Lipton R. B. (2018). Reliability and validity of ambulatory cognitive assessments. Assessment 25, 14–30. doi: 10.1177/1073191116643164, PMID: 27084835 PMC5690878

[ref85] Stenfors C. U. D. Jonsdottir I. H. Magnusson Hanson L. L. Theorell T. (2017). Associations between systemic pro-inflammatory markers, cognitive function and cognitive complaints in a population-based sample of working adults. J. Psychosom. Res. 96, 49–59. doi: 10.1016/j.jpsychores.2017.03.010, PMID: 28545793

[ref86] Tillisch K. Labus J. Kilpatrick L. Jiang Z. Stains J. Ebrat B.. (2013). Consumption of fermented milk product with probiotic modulates brain activity. Gastroenterology 144:7. doi: 10.1053/j.gastro.2013.02.043, PMID: 23474283 PMC3839572

[ref87] Turgunov Y. Ogizbayeva A. Shakeyev K. Mugazov M. Akhmaltdinova L. Nuraly S.. (2024). The dynamics of the lipopolysaccharide-binding protein (LBP) level in assessing the risk of adverse outcomes in operated colorectal cancer patients. Asian J. Surg. 47, 3435–3441. doi: 10.1016/j.asjsur.2023.08.101 37652762

[ref88] Turner M. L. Engle R. W. (1989). Is working memory capacity task dependent? J. Mem. Lang. 28, 127–154. doi: 10.1016/0749-596X(89)90040-5

[ref89] U.S. Census Bureau (2023). America is getting older. Available at: https://www.census.gov/newsroom/press-releases/2023/population-estimates-characteristics.html#:~:text=JUNE%2022%2C20202320%E28094%20The%20nation's,of%20the%20population%20is%20younger (Accessed October 15, 2023).

[ref90] van den Boogaard M. Ramakers B. P. van Alfen N. van der Werf S. P. Fick W. F. Hoedemaekers C. W.. (2010). Endotoxemia-induced inflammation and the effect on the human brain. Crit. Care 14:R81. doi: 10.1186/cc9001, PMID: 20444270 PMC2911704

[ref91] Vázquez-Martínez E. R. García-Gómez E. Camacho-Arroyo I. González-Pedrajo B. (2018). Sexual dimorphism in bacterial infections. Biol. Sex Differ. 9:1. doi: 10.1186/s13293-018-0187-5 29925409 PMC6011518

[ref92] Verhaeghen P. Basak C. (2005). Ageing and switching of the focus of attention in working memory: results from a modified N-Back task. Q. J. Exp. Psychol. A 58, 134–154. doi: 10.1080/02724980443000241 15881295

[ref93] Wang L. M. Wu Q. Kirk R. A. Horn K. P. Salem A. H. E. Hoffman J. M.. (2018). Lipopolysaccharide endotoxemia induces amyloid-β and p-tau formation in the rat brain. Am. J. Nucl. Med. Mol. Imaging 8, 86–99. 29755842 PMC5944824

[ref94] Weckesser L. J. Plessow F. Pilhatsch M. Muehlhan M. Kirschbaum C. Miller R. (2014). Do venepuncture procedures induce cortisol responses? A review, study, and synthesis for stress research. Psychoneuroendocrinology 46, 88–99. doi: 10.1016/j.psyneuen.2014.04.012 24882161

[ref96] World Health Organization (2023). Dementia. Available at: https://www.who.int/news-room/fact-sheets/detail/dementia (Accessed October 10, 2023).

[ref97] Wurfel M. M. Hailman E. Wright S. D. (1995). Soluble CD14 acts as a shuttle in the neutralization of lipopolysaccharide (LPS) by LPS-binding protein and reconstituted high density lipoprotein. J. Exp. Med. 181, 1743–1754. doi: 10.1084/jem.181.5.1743 7536794 PMC2191991

[ref98] Yao Z. Mates J. M. Cheplowitz A. M. Hammer L. P. Maiseyeu A. Phillips G. S.. (2016). Blood-borne lipopolysaccharide is rapidly eliminated by liver sinusoidal endothelial cells via high density lipoprotein. J. Immunol. 197, 2390–2399. doi: 10.4049/jimmunol.1600702, PMID: 27534554 PMC5010928

[ref99] Yarandi S. S. Peterson D. A. Treisman G. J. Moran T. H. Pasricha P. J. (2016). Modulatory effects of gut microbiota on the central nervous system: how gut could play a role in neuropsychiatric health and diseases. J. Neurogastroenterol. Motil. 22, 201–212. doi: 10.5056/jnm15146, PMID: 27032544 PMC4819858

[ref100] Yuen E. Y. Liu W. Karatsoreos I. N. Feng J. McEwen B. S. Yan Z. (2009). Acute stress enhances glutamatergic transmission in prefrontal cortex and facilitates working memory. Proc. Natl. Acad. Sci. USA 106, 14075–14079. doi: 10.1073/pnas.0906791106 19666502 PMC2729022

[ref102] Zaman G. S. Zaman F. (2015). Relationship between postprandial endotoxemia in nonobese postmenopausal women and diabetic nonobese postmenopausal women. J. Nat. Sci. Bio Med. 6, 89–93. doi: 10.4103/0976-9668.149098, PMID: 25810642 PMC4367076

[ref103] Zhang Y. Hedo R. Rivera A. Rull R. Richardson S. Tu X. M. (2019). Post hoc power analysis: is it an informative and meaningful analysis? Gen. Psych. 32:e100069. doi: 10.1136/gpsych-2019-100069, PMID: 31552383 PMC6738696

