# Supplementary material for: Cognotoxemia: endotoxemia and gender predict changes in working memory performance in healthy adults
Source: Front Neurosci. 2024 Nov 6;18:1453325. doi: 10.3389/fnins.2024.1453325 (PMC11577790; doi:10.3389/fnins.2024.1453325)
Supplement: Supplementary file 1 [file Data_Sheet_1.docx]

**Supplementary Materials for:**

**Cognotoxemia : Endotoxemia and Gender Predict Changes in Working Memory Performance in Healthy Adults**

Sally McDonnell^1^, Jennifer E. Graham-Engeland^2,3^, Martin J. Sliwinski^3,4^, Christopher G. Engeland^2,3,5^, Erik L. Knight^1*^

1. University of Colorado Boulder, Department of Psychology & Neuroscience
2. Pennsylvania State University, Biobehavioral Health
3. Pennsylvania State University, Center for Healthy Aging
4. Pennsylvania State University, Human Development and Family Studies
5. Pennsylvania State University, Ross & Carol Nese College of Nursing

*Contact: erik.knight@colorado.edu

**Supplementary Methods**

The main document and analyses utilize a composite measure of working memory, computed from participants strict scores on the Operation Span, Counting Span, and Backward Letter Span tasks. Here we report the findings for each individual working memory task. We also include findings from the other cognitive tasks in the study: the lab-based AVLT and the ecological cognitive tasks (Spatial Working Memory, N-back, and Symbol Search). Both the Spatial Working Memory and Symbol Search task are reported as error scores, while the N-back includes an overall accuracy score and an inverse efficiency score. The same two analytical approaches were employed for these outputs as in the main document (i.e. a prospective growth curve approach and a coupling approach). Age, gender, race, ethnicity, Wave 1 BMI, and education were included as covariates.

In addition to exploring gender as a moderator, we also utilize a Helmert contrast code to explore differences between pre- vs. post-menopausal women. The methodology employed for this analysis is further described in the main document.

**Supplemental Results**

**Prospective Changes in Individual Working Memory Tasks**

***Operation Span***

Levels of LBP:sCD14 at baseline do not prospectively predict changes in Operation Span performance across time (Time_Linear_ x Endotoxemia, B = -0.25, [-1.32 – 0.81], t(187.14) = -0.47, p = 0.64, Table S5). Individuals with relative low, mean, or high levels of endotoxemia do not demonstrate improvements on the Operation Span task over the span of the three waves, and their lack of improvements are not significantly different from each other.


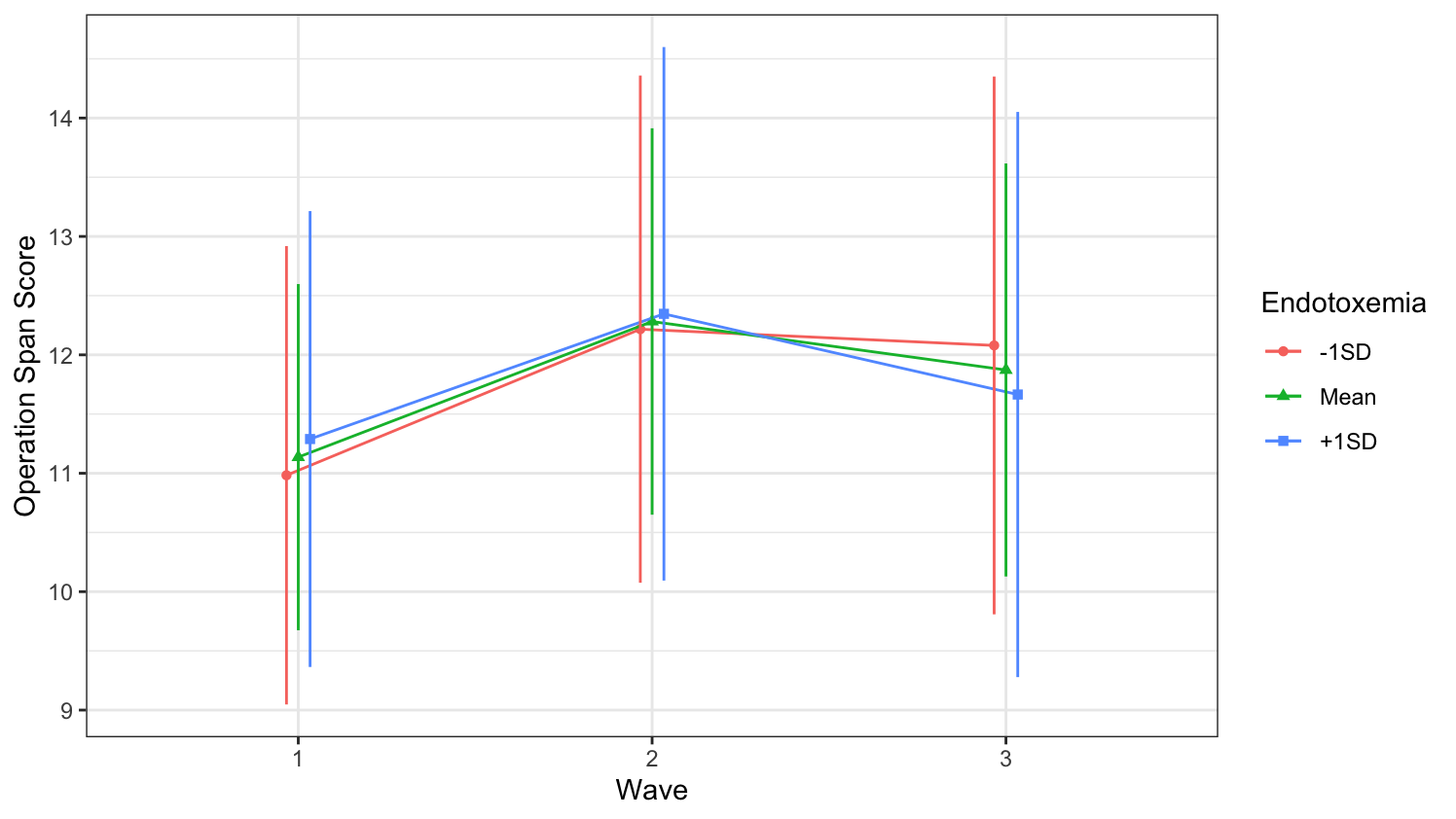


Figure S1: Baseline endotoxemia levels does not predict change (or lack thereof) in Operation Span task performance.

***Counting Span***

Endotoxemia levels at baseline predict changes in Counting Span score across time (Time_Linear_ x Endotoxemia, B = -2.21, [-4.24, -0.19], t(94.12) = -2.15, p = 0.03, Table S5). This relationship mirrors the prospective composite working memory finding reported in the main document. Individuals with relative low and mean levels of endotoxemia display improvements in Counting Span performance across time (simple slope at -1SD endotoxemia, B = 5.27, t(98.32) = 3.63, p = 0.0004; at mean, B = 3.06, t(98.81) = 2.90, p = 0.005). Individuals with higher levels of endotoxemia display no improvements on the Counting Span task across time (simple slope +1SD endotoxemia, B = 0.84, t(94.75) = 0.56, p = 0.57). This relationship was not moderated by age, sex, race, ethnicity, or education.


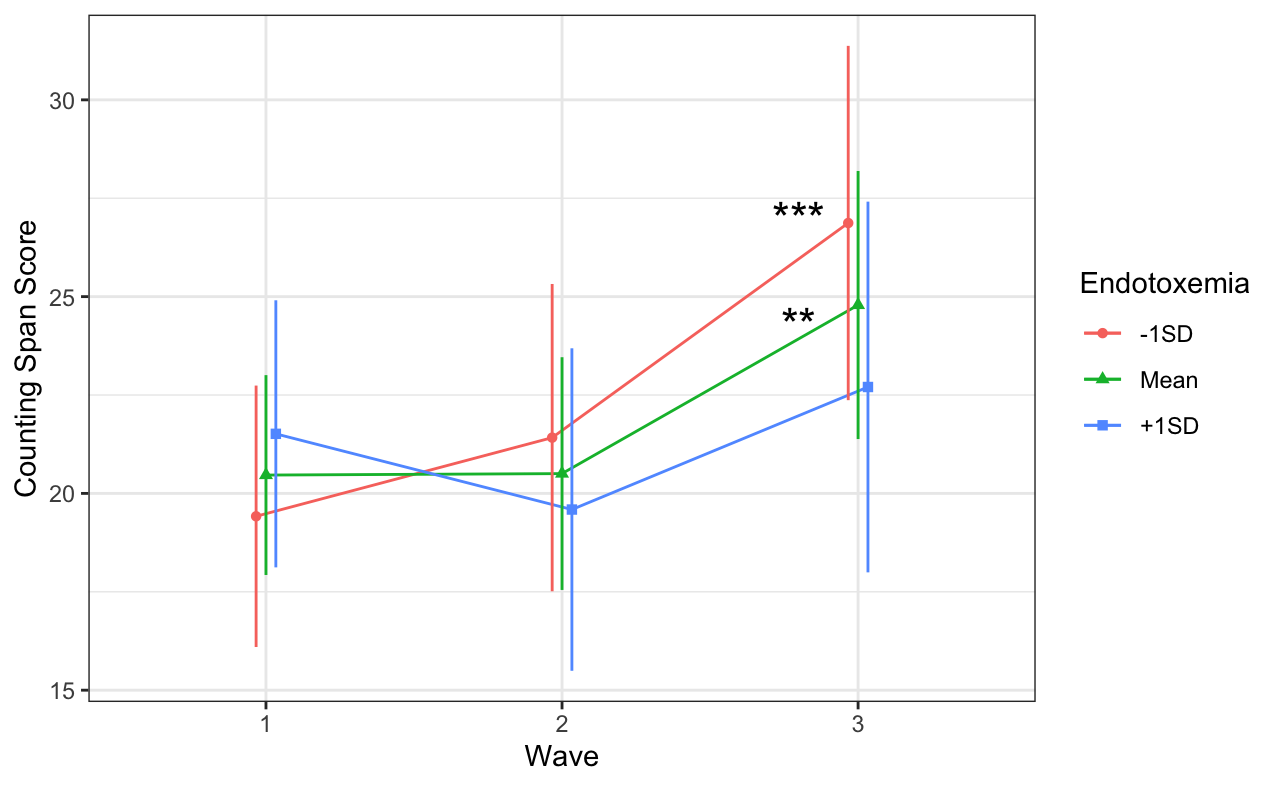


Figure S2: Baseline endotoxemia levels predict changes in Counting Span score. across time.

***Backwards Letter Span***

Endotoxemia at baseline does not seem to predict changes in Backwards Letter Span Score across time (Time_Linear_ x Endotoxemia, B = -0.58, [-1.46, 0.31], t(180.55) =  -1.28, p = 0.20, Table S5). Similar to the Operation Span task, there are not significant practice effects on the BLS task overall. However, individuals with low levels of endotoxemia display a significant improvement in BLS performance over time, though this cannot be attributed to their endotoxemia levels (B = 1.42, t(180.88) = 2.24, p = 0.03). These improvements were not shown by individuals with mean or high levels of endotoxemia (mean, B = 0.85, t(180.59) = 1.83, p = 0.07; +1SD, B = 0.27, t(180.14) = 0.41, p = 0.68) To reiterate, these differences in practice effects (or lack thereof) seen at different levels of endotoxemia are not explained by endotoxemia itself, so the differences displayed in the subsequent figure should be interpreted with caution.


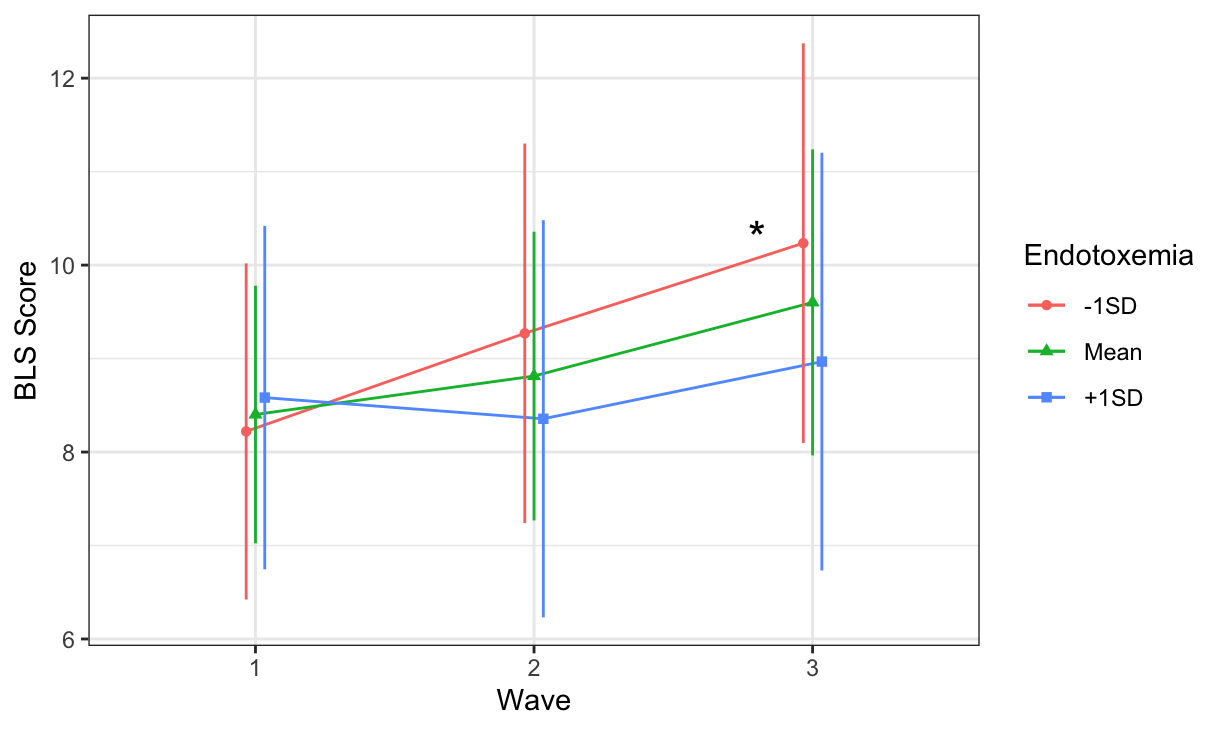


Figure S3: Baseline endotoxemia levels does not predict change in Backwards Letter Span score.

**Coupling of Endotoxemia and Individual Working Memory Tasks: Moderated By Gender**

***Operation Span***

There was no significant coupling relationship between endotoxemia and Operation Span Performance. However, a significant interaction was seen between gender and endotoxemia in the multi-level coupling model for Operation Span (Endotoxemia x Gender: B = 2.57, [0.63 – 4.50], t(120.69) = 2.57, p = 0.01, Table S9). Simple slope analyses determined a non-significant, positive association for men (B = 1.68, t(157.23) = 1.85, p = 0.066). For women, a nonsignificant negative association was observed (B = -0.88, t(37.74) = -1.63, p = 0.11).

***Counting Span***

There was a not significant coupling relationship between endotoxemia and Counting Span Performance. However, there was again an interaction between gender and endotoxemia (Endotoxemia x Gender: B = 4.67, [1.02 – 8.32], t(147.32) = 2.52, p = 0.012, Table S9). Simple slope analyses reveal that men have a significant, positive association between endotoxemia and Counting Span performance (B = 3.62, t(173.93) = 2.10, p = 0.037), while women display a nonsignificant, negative association (B = -1.05, t(88.79) = -0.98, p = 0.33).

***Backwards Letter Span***

There was no significant coupling association between BLS performance and endotoxemia. There was again an interaction between gender and endotoxemia in this multi-level coupling analysis for Backwards Letter Span (B = 2.42, [0.68 – 4.15], t(173.55) = 2.74, p = 0.007, Table S9). Similar to the results seen in the Counting Span task, men had a significant positive association (B = 1.72, t(202.64) = 2.08, p = 0.039), while women displayed a nonsignificant, negative association between endotoxemia and BLS performance (B = -0.69, t(122.27) = -1.35, p = 0.18).

**
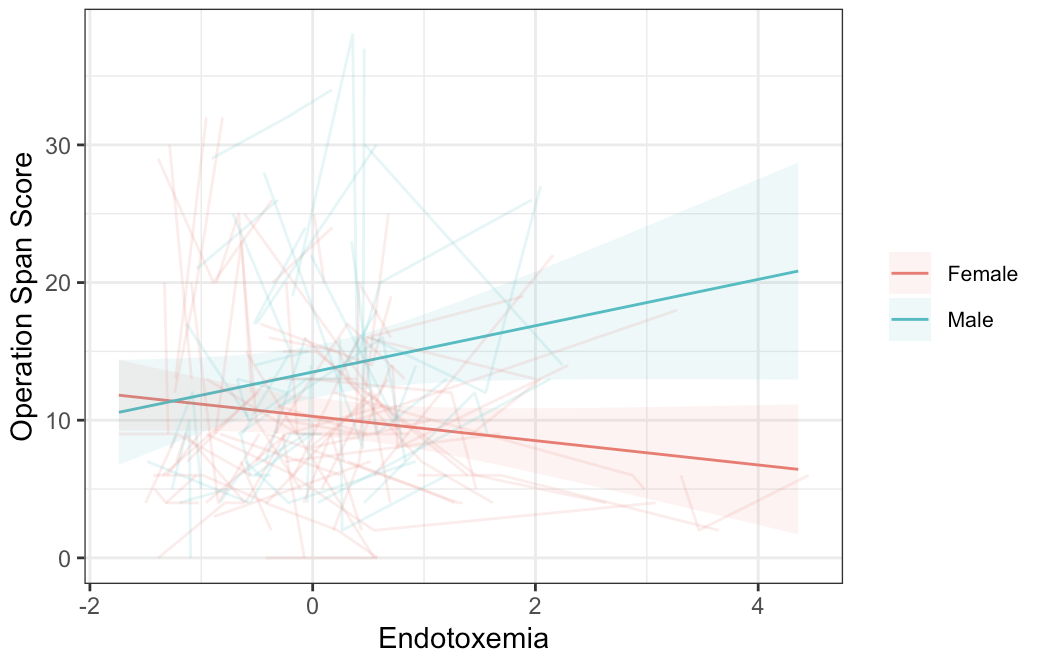
**

Figure S4: The endotoxemia and operation span performance is coupled across time differently for men and women, though simple slopes are nonsignificant. Dark lines represent the fixed effects for men and women. Lighter lines represent each individual participant’s correlation.


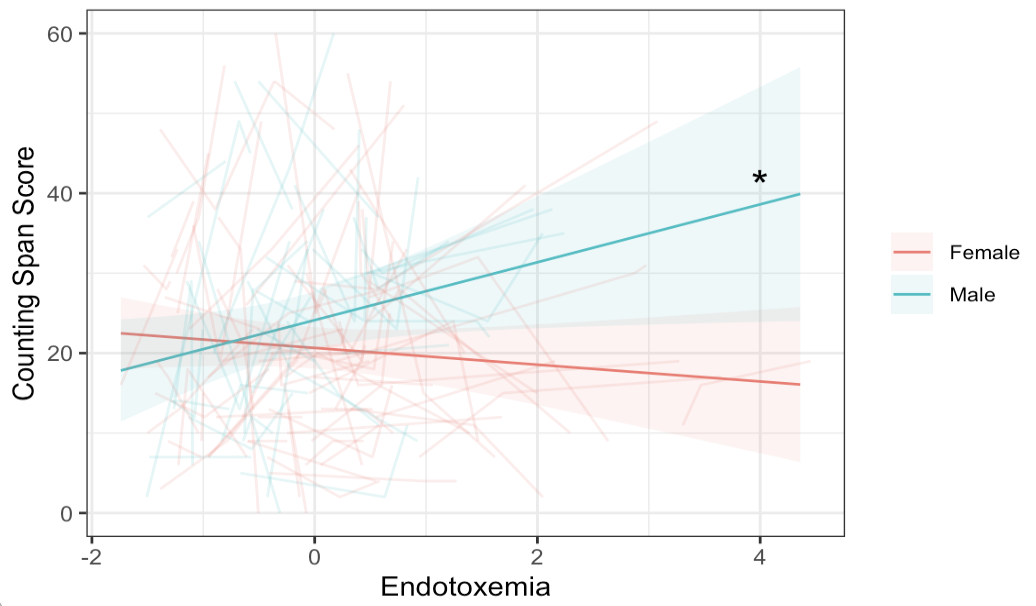


Figure S5: The endotoxemia and counting span performance is coupled across time differently for men and women. Dark lines represent the fixed effects for men and women. Lighter lines represent each individual participant’s correlation. *Simple slopes, p< .05


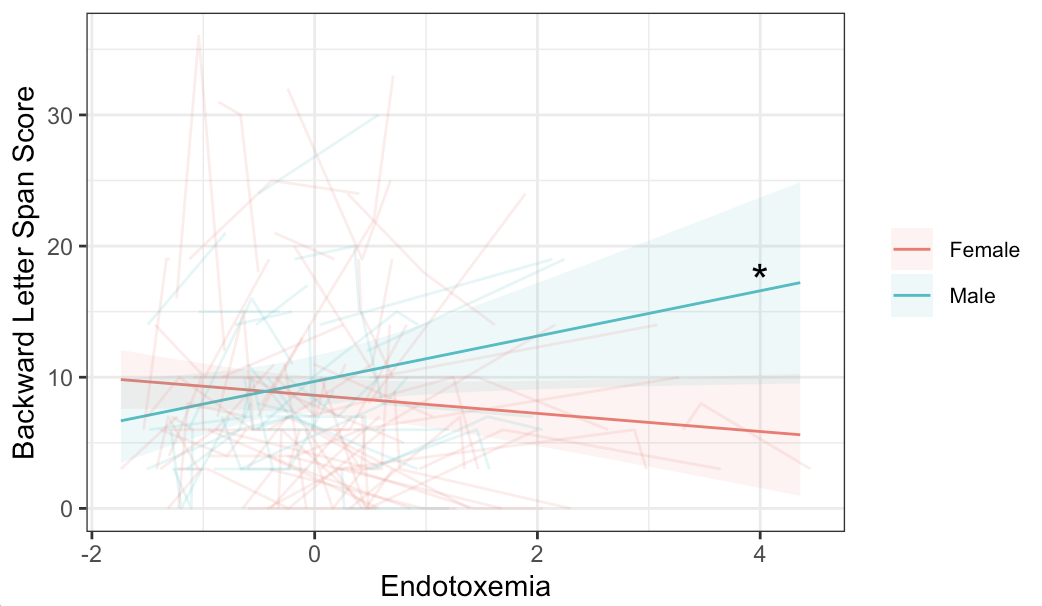


Figure S6: The endotoxemia and backward letter span performance is coupled across time differently for men and women. Dark lines represent the fixed effects for men and women. Lighter lines represent each individual participant’s correlation. *Simple slopes, p< .05

**Prospective Working Memory Changes: Differences for Pre- vs. Post-Menopausal Women**

In the main document, we report a differential finding for postmenopausal women compared to premenopausal women in the relationship between endotoxemia and prospective changes in spatial working memory performance (Time_Linear_ x Pre vs. Post-Menopause x Endotoxemia: B = 0.055, [0.005, 0.105], t(93.13) = 2.12, p = 0.037, Table S12). For post-menopausal women only, lower relative endotoxemia was associated with improved performance over time (i.e., fewer errors) (B = -0.18, *t*(87.29), = -3.31, *p* = 0.001), whereas higher levels of endotoxemia did not exhibit this association on the spatial working memory task (B = -0.015, *t*(94.10) = -0.23, *p* = 0.82). These differential practice effects were not evident for pre-menopausal women or men. This relationship is visualized in Figure S7.

Though this finding is preliminary and needs to be further explored in a larger sample of post-menopausal women, it provides initial evidence to support the specific importance of studying the effects of endotoxemia on cognitive performance in peri- and post-menopausal women. Prior work has indicated that there is a decline in epithelial barrier integrity with age, and for postmenopausal women, it is thought that this increased permeability is, at least in part, a result of declining estrogen levels (Bake et al., 2009; Cipolla et al., 2009; Wilson et al., 2008). It is possible that this change in epithelial barrier permeability post-menopause could accentuate the cognitive effects of peripheral responses to endotoxin and explain the results seen here, however more work needs to be done to substantiate such a hypothesis.

**Wave 1 Cross-sectional Analysis**


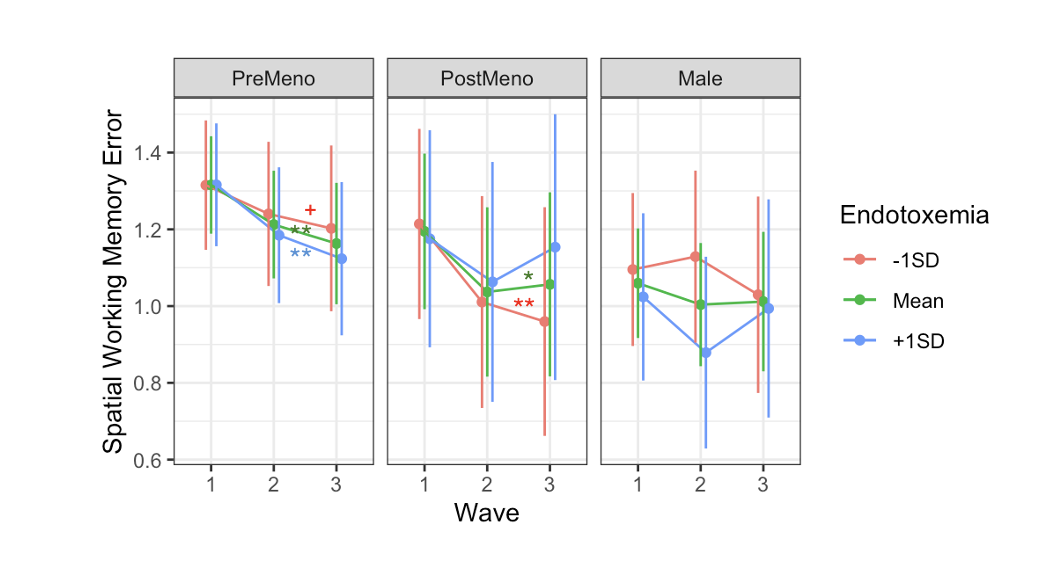


Figure S7: Endotoxemia prospectively predicts changes in spatial working memory differently for pre- and post-menopausal women. Linear effects, +*p* < .1; **p* < .05, ***p* = .001. Significant quadratic effects are not marked on the figure, but include Men +1SD (p = .02) and Post-Menopausal Women at mean levels (p = .03).

Additionally, we also explored whether there were cross-sectional associations between endotoxemia and composite working memory performance evident solely in Wave 1. This analysis revealed similar patterns to the longitudinal coupling analysis. There was no main effect of endotoxemia correlating with working in Wave 1 (p = 0.61)  and the relationship between endotoxemia and working memory performance was moderated by gender (B = 0.42, [0.12 – 0.72], t(152) = 2.80, p = 0.006, Table S17, Figure S8). For men, there was a significant, positive association of endotoxemia and working memory performance in Wave 1 (B = 0.35, t(152) = 2.64, p = 0.009); for women, there was a nonsignificant, negative association (B = -0.07, t(153) = -0.90, p = 0.37).


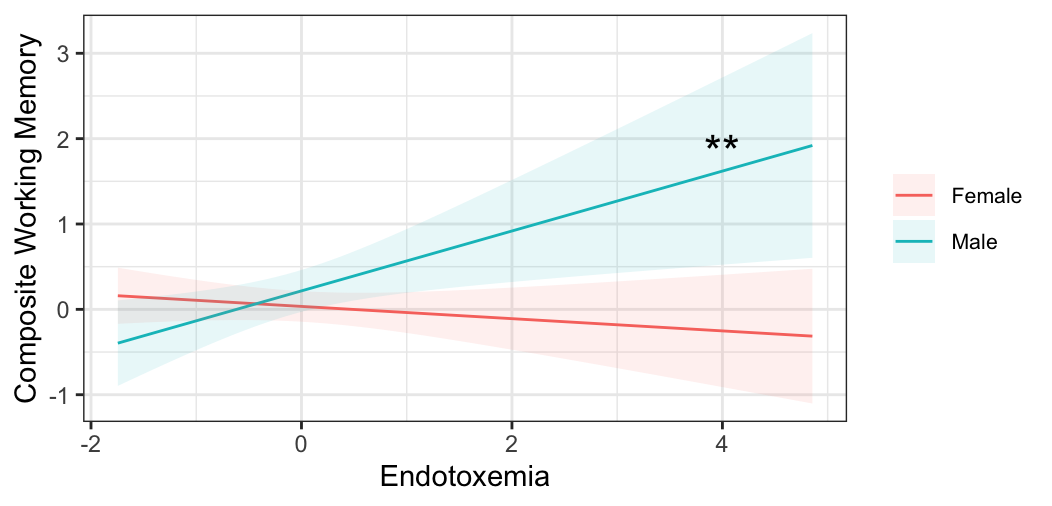


Figure S8: The association between endotoxemia and composite working memory performance in Wave 1 is different for men and women. **Simple slopes, p < 0.01

**Tables**

**Table S1: Correlations between Laboratory Working Memory Tasks**

|  | *OS* | *CS* | *BLS* |
| --- | --- | --- | --- |
| *OS* | - |  |  |
| *CS* | 0.700^***^ | - |  |
| *BLS* | 0.519^***^ | 0.569^***^ | - |
| ***p<.001* | | | |

**Table S2: Gender Differences in Endotoxemia Across All Waves of Data**

|  | **LBP:CD14** | | |
| --- | --- | --- | --- |
| *Predictors* | *Estimates* | *CI* | *p* |
| (Intercept) | -1.13 | -1.91 – -0.34 | **0.005** |
| Gender | -0.01 | -0.27 – 0.26 | 0.947 |
| Wave 1 BMI | 0.05 | 0.03 – 0.07 | **<0.001** |
| Race | -0.15 | -0.45 – 0.16 | 0.352 |
| Ethnicity | -0.22 | -0.57 – 0.13 | 0.210 |
| Education | 0.06 | -0.19 – 0.31 | 0.612 |
| Age | -0.01 | -0.02 – 0.00 | 0.155 |
| **Random Effects** | | | |
| σ^2^ | 0.43 | | |
| τ_00_ _Id_ | 0.40 | | |
| ICC | 0.48 | | |
| N _Id_ | 162 | | |
| Observations | 350 | | |
| Marginal R^2^ / Conditional R^2^ | 0.173 / 0.573 | | |

**Table S3: Gender Differences in Performance on Laboratory Cognitive Tasks Across All Data**

|  | **Operation Span** | | | **Counting Span** | | | **Backwards Letter Span** | | | **AVLT** | | |
| --- | --- | --- | --- | --- | --- | --- | --- | --- | --- | --- | --- | --- |
| *Predictors* | *Estimates* | *CI* | *p* | *Estimates* | *CI* | *p* | *Estimates* | *CI* | *p* | *Estimates* | *CI* | *p* |
| (Intercept) | 16.51 | 10.01 – 23.01 | **<0.001** | 30.11 | 18.22 – 42.01 | **<0.001** | 16.47 | 10.09 – 22.84 | **<0.001** | 10.17 | 8.08 – 12.26 | **<0.001** |
| Gender | 3.14 | 0.95 – 5.34 | **0.005** | 2.80 | -1.22 – 6.83 | 0.171 | 0.94 | -1.22 – 3.09 | 0.394 | -0.17 | -0.87 – 0.54 | 0.639 |
| Wave 1 BMI | -0.06 | -0.19 – 0.07 | 0.341 | -0.01 | -0.25 – 0.23 | 0.964 | -0.01 | -0.14 – 0.11 | 0.829 | -0.01 | -0.05 – 0.03 | 0.720 |
| Race | -1.08 | -3.63 – 1.47 | 0.403 | -0.24 | -4.94 – 4.45 | 0.918 | -1.72 | -4.25 – 0.80 | 0.180 | -0.67 | -1.49 – 0.16 | 0.113 |
| Ethnicity | -2.23 | -5.14 – 0.67 | 0.132 | -3.76 | -9.06 – 1.54 | 0.164 | -0.91 | -3.76 – 1.93 | 0.528 | -0.70 | -1.64 – 0.24 | 0.143 |
| Education | 2.48 | 0.41 – 4.56 | **0.019** | 3.07 | -0.74 – 6.89 | 0.114 | 1.10 | -0.94 – 3.14 | 0.290 | 0.89 | 0.22 – 1.56 | **0.009** |
| Age | -0.09 | -0.18 – 0.00 | 0.051 | -0.21 | -0.38 – -0.04 | **0.015** | -0.16 | -0.25 – -0.07 | **0.001** | -0.03 | -0.06 – 0.00 | 0.065 |
| **Random Effects** | | | | | | | | | | | | |
| σ^2^ | 27.25 | | | 93.48 | | | 19.61 | | | 2.66 | | |
| τ_00_ | 26.29 _Id_ | | | 93.24 _Id_ | | | 30.86 _Id_ | | | 3.01 _Id_ | | |
| ICC | 0.49 | | | 0.50 | | | 0.61 | | | 0.53 | | |
| N | 154 _Id_ | | | 160 _Id_ | | | 162 _Id_ | | | 162 _Id_ | | |
| Observations | 326 | | | 338 | | | 343 | | | 342 | | |
| Marginal R^2^ / Conditional R^2^ | 0.092 / 0.538 | | | 0.052 / 0.525 | | | 0.065 / 0.637 | | | 0.057 / 0.558 | | |

**Table S4: Gender Differences in Performance on Ambulatory Cognitive Tasks Across All Data**

|  | **Symbol Search** | | | **N-Back** | | | **N-Back (IES)** | | | **Spatial WM Error** | | |
| --- | --- | --- | --- | --- | --- | --- | --- | --- | --- | --- | --- | --- |
| *Predictors* | *Estimates* | *CI* | *p* | *Estimates* | *CI* | *p* | *Estimates* | *CI* | *p* | *Estimates* | *CI* | *p* |
| (Intercept) | 7.34 | 7.12 – 7.56 | **<0.001** | 0.89 | 0.78 – 1.00 | **<0.001** | 5.97 | 5.68 – 6.26 | **<0.001** | 0.88 | 0.40 – 1.37 | **<0.001** |
| Gender | 0.01 | -0.07 – 0.08 | 0.878 | 0.01 | -0.02 – 0.05 | 0.476 | -0.01 | -0.11 – 0.09 | 0.883 | -0.16 | -0.32 – 0.00 | 0.057 |
| Wave 1 BMI | 0.00 | -0.00 – 0.01 | 0.077 | 0.00 | -0.00 – 0.00 | 0.889 | 0.00 | -0.00 – 0.01 | 0.418 | 0.01 | -0.00 – 0.02 | 0.092 |
| Race | -0.01 | -0.10 – 0.08 | 0.851 | -0.04 | -0.08 – 0.01 | 0.108 | -0.05 | -0.16 – 0.07 | 0.443 | 0.10 | -0.09 – 0.29 | 0.316 |
| Ethnicity | -0.04 | -0.14 – 0.05 | 0.373 | -0.04 | -0.09 – 0.01 | 0.147 | -0.01 | -0.14 – 0.12 | 0.908 | -0.01 | -0.22 – 0.21 | 0.953 |
| Education | -0.05 | -0.12 – 0.02 | 0.186 | 0.04 | 0.00 – 0.07 | **0.049** | 0.01 | -0.08 – 0.10 | 0.832 | -0.17 | -0.33 – -0.02 | **0.029** |
| Age | 0.01 | 0.01 – 0.01 | **<0.001** | -0.00 | -0.00 – 0.00 | 0.191 | 0.01 | 0.01 – 0.02 | **<0.001** | 0.00 | -0.00 – 0.01 | 0.489 |
| **Random Effects** | | | | | | | | | | | | |
| σ^2^ | 0.01 | | | 0.00 | | | 0.05 | | | 0.04 | | |
| τ_00_ | 0.04 _Id_ | | | 0.01 _Id_ | | | 0.06 _Id_ | | | 0.22 _Id_ | | |
| ICC | 0.83 | | | 0.85 | | | 0.58 | | | 0.86 | | |
| N | 162 _Id_ | | | 162 _Id_ | | | 162 _Id_ | | | 162 _Id_ | | |
| Observations | 355 | | | 355 | | | 355 | | | 355 | | |
| Marginal R^2^ / Conditional R^2^ | 0.178 / 0.858 | | | 0.044 / 0.857 | | | 0.142 / 0.636 | | | 0.083 / 0.868 | | |

**Table S5: Prospective Models for Laboratory Cognitive Tasks**

|  | **Operation Span** | | | **Counting Span** | | | **Backwards Letter Span** | | | **AVLT** | | |
| --- | --- | --- | --- | --- | --- | --- | --- | --- | --- | --- | --- | --- |
| *Predictors* | *Estimates* | *CI* | *p* | *Estimates* | *CI* | *p* | *Estimates* | *CI* | *p* | *Estimates* | *CI* | *p* |
| (Intercept) | 16.64 | 10.03 – 23.25 | **<0.001** | 30.32 | 18.25 – 42.40 | **<0.001** | 16.64 | 10.13 – 23.14 | **<0.001** | 10.36 | 8.27 – 12.45 | **<0.001** |
| Time Linear | 0.52 | -0.58 – 1.62 | 0.351 | 3.06 | 0.98 – 5.13 | **0.004** | 0.85 | -0.06 – 1.76 | 0.068 | 0.26 | -0.06 – 0.59 | 0.115 |
| Time Quadratic | -0.63 | -1.69 – 0.42 | 0.238 | 1.73 | -0.04 – 3.51 | 0.056 | 0.15 | -0.73 – 1.04 | 0.733 | -0.37 | -0.70 – -0.05 | **0.023** |
| Wave 1 LBP:CD14 | 0.00 | -1.13 – 1.13 | 0.995 | -0.65 | -2.83 – 1.54 | 0.559 | -0.30 | -1.43 – 0.83 | 0.598 | 0.23 | -0.13 – 0.58 | 0.216 |
| Age | -0.09 | -0.19 – 0.00 | 0.054 | -0.22 | -0.39 – -0.05 | **0.013** | -0.16 | -0.25 – -0.07 | **0.001** | -0.02 | -0.05 – 0.00 | 0.100 |
| Wave 1 BMI | -0.06 | -0.20 – 0.08 | 0.370 | 0.01 | -0.25 – 0.27 | 0.922 | -0.01 | -0.15 – 0.13 | 0.906 | -0.02 | -0.06 – 0.03 | 0.454 |
| Gender | 3.13 | 0.93 – 5.33 | **0.006** | 2.68 | -1.33 – 6.69 | 0.190 | 0.96 | -1.21 – 3.13 | 0.384 | -0.17 | -0.87 – 0.52 | 0.626 |
| Race | -1.06 | -3.62 – 1.50 | 0.415 | -0.05 | -4.75 – 4.66 | 0.984 | -1.72 | -4.27 – 0.82 | 0.183 | -0.60 | -1.41 – 0.22 | 0.151 |
| Ethnicity | -2.14 | -5.07 – 0.79 | 0.151 | -3.89 | -9.20 – 1.41 | 0.150 | -0.94 | -3.82 – 1.93 | 0.520 | -0.59 | -1.52 – 0.34 | 0.211 |
| Education | 2.48 | 0.40 – 4.56 | **0.020** | 2.98 | -0.83 – 6.79 | 0.125 | 1.06 | -0.99 – 3.12 | 0.309 | 0.86 | 0.20 – 1.52 | **0.010** |
| Time Linear x Wave 1 LBP:CD14 | -0.25 | -1.32 – 0.81 | 0.640 | -2.21 | -4.24 – -0.19 | **0.032** | -0.58 | -1.46 – 0.31 | 0.203 | 0.29 | -0.03 – 0.61 | 0.080 |
| Time Quadratic x Wave 1 LBP:CD14 | -0.07 | -1.14 – 0.99 | 0.890 | 0.32 | -1.44 – 2.09 | 0.719 | 0.19 | -0.70 – 1.08 | 0.677 | -0.08 | -0.41 – 0.24 | 0.609 |
| **Random Effects** | | | | | | | | | | | | |
| σ^2^ | 27.52 | | | 75.35 | | | 19.41 | | | 2.59 | | |
| τ_00_ | 26.31 _Id_ | | | 109.43 _Id_ | | | 31.55 _Id_ | | | 2.84 _Id_ | | |
| τ_11_ | 0.00 _Id.burst_L1_ | | | 24.77 _Id.burst_L1_ | | | 0.01 _Id.burst_L1_ | | | 0.02 _Id.burst_L1_ | | |
| ρ_01_ | 1.00 _Id_ | | | 0.38 _Id_ | | | 1.00 _Id_ | | | -1.00 _Id_ | | |
| ICC |  | | | 0.59 | | |  | | |  | | |
| N | 154 _Id_ | | | 160 _Id_ | | | 162 _Id_ | | | 162 _Id_ | | |
| Observations | 326 | | | 338 | | | 343 | | | 342 | | |
| Marginal R^2^ / Conditional R^2^ | 0.172 / NA | | | 0.075 / 0.623 | | | 0.167 / NA | | | 0.154 / NA | | |

**Table S6: Prospective Models for Ambulatory Cognitive Tasks**

|  | **Symbol Search** | | | **N-Back** | | | **N-Back (IES)** | | | **Spatial WM Error** | | |
| --- | --- | --- | --- | --- | --- | --- | --- | --- | --- | --- | --- | --- |
| *Predictors* | *Estimates* | *CI* | *p* | *Estimates* | *CI* | *p* | *Estimates* | *CI* | *p* | *Estimates* | *CI* | *p* |
| (Intercept) | 7.32 | 7.10 – 7.54 | **<0.001** | 0.89 | 0.78 – 1.00 | **<0.001** | 5.92 | 5.64 – 6.21 | **<0.001** | 0.92 | 0.44 – 1.39 | **<0.001** |
| Time Linear | -0.06 | -0.08 – -0.04 | **<0.001** | 0.03 | 0.02 – 0.04 | **<0.001** | -0.23 | -0.26 – -0.20 | **<0.001** | -0.09 | -0.13 – -0.05 | **<0.001** |
| Time Quadratic | 0.01 | -0.00 – 0.03 | 0.112 | -0.01 | -0.01 – -0.00 | **0.044** | 0.05 | 0.02 – 0.07 | **0.001** | 0.03 | -0.00 – 0.06 | 0.059 |
| Wave 1 LBP:CD14 | -0.01 | -0.04 – 0.03 | 0.789 | -0.01 | -0.02 – 0.01 | 0.485 | -0.02 | -0.07 – 0.03 | 0.475 | -0.02 | -0.10 – 0.07 | 0.702 |
| Age | 0.01 | 0.01 – 0.01 | **<0.001** | -0.00 | -0.00 – 0.00 | 0.281 | 0.01 | 0.01 – 0.01 | **<0.001** | 0.00 | -0.00 – 0.01 | 0.330 |
| Wave 1 BMI | 0.00 | -0.00 – 0.01 | 0.081 | 0.00 | -0.00 – 0.00 | 0.819 | 0.00 | -0.00 – 0.01 | 0.235 | 0.01 | -0.00 – 0.02 | 0.197 |
| Gender | 0.01 | -0.07 – 0.08 | 0.877 | 0.01 | -0.03 – 0.05 | 0.640 | -0.02 | -0.11 – 0.08 | 0.751 | -0.22 | -0.37 – -0.06 | **0.007** |
| Race | -0.01 | -0.10 – 0.07 | 0.779 | -0.03 | -0.08 – 0.01 | 0.129 | -0.07 | -0.18 – 0.05 | 0.246 | 0.07 | -0.12 – 0.25 | 0.460 |
| Ethnicity | -0.05 | -0.14 – 0.05 | 0.338 | -0.03 | -0.08 – 0.02 | 0.208 | -0.03 | -0.15 – 0.10 | 0.647 | -0.03 | -0.24 – 0.18 | 0.783 |
| Education | -0.05 | -0.12 – 0.02 | 0.154 | 0.03 | -0.01 – 0.06 | 0.132 | 0.02 | -0.07 – 0.11 | 0.692 | -0.17 | -0.32 – -0.02 | **0.025** |
| Time Linear x Wave 1 LBP:CD14 | -0.00 | -0.02 – 0.02 | 0.975 | 0.00 | -0.01 – 0.01 | 0.855 | -0.01 | -0.04 – 0.02 | 0.380 | 0.00 | -0.04 – 0.05 | 0.830 |
| Time Quadratic x Wave 1 LBP:CD14 | 0.01 | -0.01 – 0.02 | 0.280 | -0.00 | -0.01 – 0.00 | 0.373 | 0.01 | -0.01 – 0.04 | 0.297 | 0.03 | -0.01 – 0.06 | 0.104 |
| **Random Effects** | | | | | | | | | | | | |
| σ^2^ | 0.01 | | | 0.00 | | | 0.02 | | | 0.02 | | |
| τ_00_ | 0.05 _Id_ | | | 0.01 _Id_ | | | 0.07 _Id_ | | | 0.24 _Id_ | | |
| τ_11_ | 0.00 _Id.burst_L1_ | | | 0.00 _Id.burst_L1_ | | | 0.00 _Id.burst_L1_ | | | 0.02 _Id.burst_L1_ | | |
| ρ_01_ | 0.32 _Id_ | | | -0.33 _Id_ | | | -0.26 _Id_ | | | 0.55 _Id_ | | |
| ICC | 0.88 | | | 0.92 | | | 0.80 | | | 0.91 | | |
| N | 162 _Id_ | | | 162 _Id_ | | | 162 _Id_ | | | 162 _Id_ | | |
| Observations | 355 | | | 355 | | | 355 | | | 355 | | |
| Marginal R^2^ / Conditional R^2^ | 0.199 / 0.903 | | | 0.062 / 0.926 | | | 0.297 / 0.858 | | | 0.093 / 0.918 | | |

**Table S7: Main Effects of Coupling Model of Laboratory Cognitive Tasks and Composite Working Memory Measure**

|  | **Composite Working Memory** | | | **Operation Span** | | | **Counting Span** | | | **Backwards Letter Span** | | | | **AVLT** | | |
| --- | --- | --- | --- | --- | --- | --- | --- | --- | --- | --- | --- | --- | --- | --- | --- | --- |
| *Predictors* | *Estimates* | *CI* | *p* | *Estimates* | *CI* | *p* | *Estimates* | *CI* | *p* | *Estimates* | *CI* | *p* | *Estimates* | | *CI* | *p* |
| (Intercept) | 1.20 | 0.41 – 1.99 | **0.003** | 17.85 | 11.63 – 24.07 | **<0.001** | 30.55 | 18.52 – 42.59 | **<0.001** | 17.89 | 11.35 – 24.42 | **<0.001** | 10.15 | | 8.07 – 12.23 | **<0.001** |
| Time Linear | 0.16 | 0.04 – 0.28 | **0.008** | 0.64 | -0.62 – 1.91 | 0.319 | 2.89 | 0.57 – 5.21 | **0.015** | 0.98 | -0.05 – 2.02 | 0.062 | 0.24 | | -0.15 – 0.63 | 0.230 |
| Time Quadratic | 0.03 | -0.07 – 0.13 | 0.575 | -0.57 | -1.68 – 0.55 | 0.320 | 1.78 | -0.24 – 3.79 | 0.084 | 0.09 | -0.77 – 0.95 | 0.840 | -0.38 | | -0.73 – -0.04 | **0.027** |
| LBP:CD14 | -0.01 | -0.13 – 0.10 | 0.800 | -0.22 | -1.25 – 0.80 | 0.666 | 0.04 | -1.92 – 1.99 | 0.970 | -0.12 | -1.07 – 0.82 | 0.797 | -0.06 | | -0.38 – 0.26 | 0.713 |
| Wave 1 BMI | -0.01 | -0.02 – 0.01 | 0.499 | -0.08 | -0.21 – 0.04 | 0.194 | -0.04 | -0.30 – 0.22 | 0.784 | -0.04 | -0.18 – 0.10 | 0.589 | -0.01 | | -0.05 – 0.04 | 0.810 |
| Gender | 0.22 | -0.05 – 0.48 | 0.106 | 3.56 | 1.46 – 5.67 | **0.001** | 2.50 | -1.47 – 6.47 | 0.217 | 0.44 | -1.73 – 2.61 | 0.688 | -0.10 | | -0.80 – 0.59 | 0.774 |
| Age | -0.02 | -0.03 – -0.01 | **0.001** | -0.09 | -0.18 – -0.00 | **0.048** | -0.20 | -0.37 – -0.03 | **0.021** | -0.17 | -0.26 – -0.07 | **<0.001** | -0.03 | | -0.06 – 0.00 | 0.073 |
| Race | -0.13 | -0.44 – 0.18 | 0.409 | -1.94 | -4.35 – 0.46 | 0.112 | 0.40 | -4.26 – 5.06 | 0.866 | -1.59 | -4.15 – 0.97 | 0.223 | -0.74 | | -1.55 – 0.08 | 0.078 |
| Ethnicity | -0.23 | -0.57 – 0.12 | 0.202 | -3.01 | -5.83 – -0.18 | **0.037** | -2.85 | -8.09 – 2.39 | 0.286 | -0.77 | -3.63 – 2.10 | 0.599 | -0.75 | | -1.70 – 0.19 | 0.115 |
| Education | 0.24 | -0.01 – 0.49 | 0.055 | 2.55 | 0.59 – 4.52 | **0.011** | 2.92 | -0.86 – 6.70 | 0.130 | 1.22 | -0.85 – 3.28 | 0.248 | 0.89 | | 0.23 – 1.54 | **0.009** |
| **Random Effects** | | | | | | | | | | | | | | | | |
| σ^2^ | 0.22 | | | 27.75 | | | 90.83 | | | 16.23 | | | | 2.63 | | |
| τ_00_ | 0.49 _Id_ | | | 21.48 _Id_ | | | 90.25 _Id_ | | | 33.57 _Id_ | | | | 2.82 _Id_ | | |
| τ_11_ | 0.01 _Id.LBP_CD14_ | | | 3.18 _Id.LBP_CD14_ | | | 0.56 _Id.LBP_CD14_ | | | 0.11 _Id.LBP_CD14_ | | | | 0.01 _Id.LBP_CD14_ | | |
| ρ_01_ | 0.11 _Id_ | | | -0.73 _Id_ | | | 0.65 _Id_ | | | 1.00 _Id_ | | | | -1.00 _Id_ | | |
| ICC | 0.70 | | | 0.47 | | | 0.50 | | | 0.67 | | | |  | | |
| N | 162 _Id_ | | | 154 _Id_ | | | 160 _Id_ | | | 162 _Id_ | | | | 161 _Id_ | | |
| Observations | 330 | | | 313 | | | 325 | | | 330 | | | | 329 | | |
| Marginal R^2^ / Conditional R^2^ | 0.100 / 0.727 | | | 0.125 / 0.536 | | | 0.062 / 0.530 | | | 0.076 / 0.699 | | | | 0.135 / NA | | |

**Table S8: Main Effects of Coupling Model for Ambulatory Cognitive Tasks**

|  | **Symbol Search** | | | **N-Back** | | | **N-Back (IES)** | | | **Spatial Working Memory Error** | | |
| --- | --- | --- | --- | --- | --- | --- | --- | --- | --- | --- | --- | --- |
| *Predictors* | *Estimates* | *CI* | *p* | *Estimates* | *CI* | *p* | *Estimates* | *CI* | *p* | *Estimates* | *CI* | *p* |
| (Intercept) | 7.32 | 7.09 – 7.54 | **<0.001** | 0.90 | 0.79 – 1.01 | **<0.001** | 5.87 | 5.61 – 6.14 | **<0.001** | 0.86 | 0.38 – 1.34 | **<0.001** |
| Time Linear | -0.07 | -0.09 – -0.04 | **<0.001** | 0.03 | 0.02 – 0.03 | **<0.001** | -0.22 | -0.26 – -0.19 | **<0.001** | -0.10 | -0.15 – -0.05 | **<0.001** |
| Time Quadratic | 0.01 | -0.01 – 0.03 | 0.204 | -0.01 | -0.02 – -0.00 | **0.034** | 0.05 | 0.02 – 0.08 | **0.001** | 0.04 | -0.00 – 0.08 | 0.052 |
| LBP:CD14 | 0.00 | -0.02 – 0.03 | 0.801 | 0.01 | -0.00 – 0.02 | 0.231 | -0.02 | -0.05 – 0.02 | 0.308 | 0.02 | -0.04 – 0.07 | 0.545 |
| Wave 1 BMI | 0.00 | -0.00 – 0.01 | 0.065 | 0.00 | -0.00 – 0.00 | 0.857 | 0.00 | -0.00 – 0.01 | 0.175 | 0.01 | -0.00 – 0.02 | 0.154 |
| Gender | 0.01 | -0.07 – 0.08 | 0.829 | 0.02 | -0.02 – 0.05 | 0.391 | -0.03 | -0.13 – 0.06 | 0.470 | -0.17 | -0.33 – -0.01 | **0.034** |
| Age | 0.01 | 0.01 – 0.01 | **<0.001** | -0.00 | -0.00 – 0.00 | 0.184 | 0.01 | 0.01 – 0.02 | **<0.001** | 0.00 | -0.00 – 0.01 | 0.381 |
| Race | -0.01 | -0.09 – 0.08 | 0.906 | -0.04 | -0.08 – 0.01 | 0.108 | -0.06 | -0.17 – 0.04 | 0.239 | 0.10 | -0.08 – 0.29 | 0.278 |
| Ethnicity | -0.05 | -0.15 – 0.05 | 0.331 | -0.03 | -0.08 – 0.02 | 0.186 | -0.02 | -0.14 – 0.10 | 0.782 | -0.01 | -0.22 – 0.20 | 0.948 |
| Education | -0.05 | -0.12 – 0.02 | 0.150 | 0.03 | -0.01 – 0.06 | 0.098 | 0.02 | -0.07 – 0.11 | 0.633 | -0.16 | -0.32 – -0.01 | **0.035** |
| **Random Effects** | | | | | | | | | | | | |
| σ^2^ | 0.01 | | | 0.00 | | | 0.02 | | | 0.03 | | |
| τ_00_ | 0.05 _Id_ | | | 0.01 _Id_ | | | 0.07 _Id_ | | | 0.22 _Id_ | | |
| τ_11_ | 0.00 _Id.LBP_CD14_ | | | 0.00 _Id.LBP_CD14_ | | | 0.00 _Id.LBP_CD14_ | | | 0.00 _Id.LBP_CD14_ | | |
| ρ_01_ | 1.00 _Id_ | | | -0.42 _Id_ | | | -0.94 _Id_ | | | 0.68 _Id_ | | |
| ICC |  | | | 0.92 | | | 0.78 | | | 0.88 | | |
| N | 162 _Id_ | | | 162 _Id_ | | | 162 _Id_ | | | 162 _Id_ | | |
| Observations | 331 | | | 331 | | | 331 | | | 331 | | |
| Marginal R^2^ / Conditional R^2^ | 0.631 / NA | | | 0.068 / 0.921 | | | 0.315 / 0.847 | | | 0.097 / 0.891 | | |

**Table S9: Coupling Model of Endotoxemia x Gender for Laboratory Based Tasks**

|  | **Operation Span** | | | **Counting Span** | | | **Backwards Letter Span** | | | | **AVLT** | | |
| --- | --- | --- | --- | --- | --- | --- | --- | --- | --- | --- | --- | --- | --- |
| *Predictors* | *Estimates* | *CI* | *p* | *Estimates* | *CI* | *p* | *Estimates* | *CI* | *p* | *Estimates* | | *CI* | *p* |
| (Intercept) | 18.15 | 12.07 – 24.23 | **<0.001** | 30.98 | 19.19 – 42.76 | **<0.001** | 18.07 | 11.69 – 24.45 | **<0.001** | 10.19 | | 8.12 – 12.26 | **<0.001** |
| Time Linear | 0.66 | -0.60 – 1.93 | 0.303 | 2.82 | 0.50 – 5.13 | **0.017** | 0.94 | -0.09 – 1.97 | 0.074 | 0.23 | | -0.16 – 0.62 | 0.244 |
| Time Quadratic | -0.52 | -1.64 – 0.60 | 0.364 | 1.92 | -0.10 – 3.95 | 0.063 | 0.16 | -0.70 – 1.02 | 0.716 | -0.38 | | -0.72 – -0.03 | **0.031** |
| LBP;CD14 | -0.88 | -1.95 – 0.18 | 0.105 | -1.05 | -3.15 – 1.05 | 0.327 | -0.69 | -1.70 – 0.32 | 0.178 | -0.12 | | -0.46 – 0.22 | 0.500 |
| Gender | 3.22 | 1.14 – 5.30 | **0.003** | 3.47 | -0.48 – 7.43 | 0.085 | 1.06 | -1.09 – 3.21 | 0.333 | -0.08 | | -0.78 – 0.61 | 0.820 |
| Age | -0.10 | -0.19 – -0.01 | **0.025** | -0.22 | -0.39 – -0.05 | **0.010** | -0.18 | -0.27 – -0.08 | **<0.001** | -0.03 | | -0.06 – 0.00 | 0.060 |
| Wave 1 BMI | -0.08 | -0.20 – 0.05 | 0.221 | -0.03 | -0.28 – 0.22 | 0.817 | -0.03 | -0.17 – 0.10 | 0.612 | -0.00 | | -0.05 – 0.04 | 0.823 |
| Race | -1.97 | -4.34 – 0.39 | 0.102 | 0.23 | -4.32 – 4.79 | 0.919 | -1.71 | -4.20 – 0.79 | 0.180 | -0.74 | | -1.55 – 0.08 | 0.077 |
| Ethnicity | -2.67 | -5.46 – 0.11 | 0.060 | -2.59 | -7.72 – 2.55 | 0.322 | -0.68 | -3.49 – 2.12 | 0.631 | -0.73 | | -1.66 – 0.21 | 0.128 |
| Education | 2.67 | 0.74 – 4.60 | **0.007** | 3.16 | -0.54 – 6.87 | 0.094 | 1.35 | -0.67 – 3.37 | 0.189 | 0.90 | | 0.24 – 1.56 | **0.007** |
| LBP:CD14 × Gender | 2.57 | 0.63 – 4.50 | **0.010** | 4.67 | 1.02 – 8.32 | **0.012** | 2.42 | 0.68 – 4.15 | **0.006** | 0.28 | | -0.33 – 0.89 | 0.373 |
| **Random Effects** | | | | | | | | | | | | | |
| σ^2^ | 28.12 | | | 91.76 | | | 16.38 | | | | 2.65 | | |
| τ_00_ | 20.57 _Id_ | | | 83.90 _Id_ | | | 31.22 _Id_ | | | | 2.77 _Id_ | | |
| τ_11_ | 2.17 _Id.LBP_CD14_ | | | 0.23 _Id.LBP_CD14_ | | | 0.04 _Id.LBP_CD14_ | | | | 0.01 _Id.LBP_CD14_ | | |
| ρ_01_ | -0.80 _Id_ | | | 1.00 _Id_ | | | 1.00 _Id_ | | | | -1.00 _Id_ | | |
| ICC | 0.45 | | |  | | |  | | | |  | | |
| N | 154 _Id_ | | | 160 _Id_ | | | 162 _Id_ | | | | 161 _Id_ | | |
| Observations | 313 | | | 325 | | | 330 | | | | 329 | | |
| Marginal R^2^ / Conditional R^2^ | 0.130 / 0.519 | | | 0.146 / NA | | | 0.238 / NA | | | | 0.137 / NA | | |

**Table S10: Coupling Model of Endotoxemia x Gender for Ambulatory Cognitive Tasks**

|  | **Symbol Search** | | | **N-Back** | | | **N-Back (IES)** | | | **Spatial WM Error** | | |
| --- | --- | --- | --- | --- | --- | --- | --- | --- | --- | --- | --- | --- |
| *Predictors* | *Estimates* | *CI* | *p* | *Estimates* | *CI* | *p* | *Estimates* | *CI* | *p* | *Estimates* | *CI* | *p* |
| (Intercept) | 7.31 | 7.09 – 7.54 | **<0.001** | 0.90 | 0.79 – 1.01 | **<0.001** | 5.87 | 5.60 – 6.14 | **<0.001** | 0.86 | 0.38 – 1.34 | **<0.001** |
| Time Linear | -0.06 | -0.09 – -0.04 | **<0.001** | 0.03 | 0.02 – 0.03 | **<0.001** | -0.22 | -0.26 – -0.19 | **<0.001** | -0.10 | -0.15 – -0.05 | **<0.001** |
| Time Quadratic | 0.01 | -0.01 – 0.03 | 0.248 | -0.01 | -0.02 – -0.00 | **0.033** | 0.05 | 0.02 – 0.08 | **0.001** | 0.04 | 0.00 – 0.08 | **0.050** |
| LBP:CD14 | 0.01 | -0.01 – 0.04 | 0.391 | 0.01 | -0.00 – 0.02 | 0.188 | -0.01 | -0.04 – 0.03 | 0.678 | 0.01 | -0.05 – 0.07 | 0.800 |
| Gender | -0.00 | -0.08 – 0.07 | 0.945 | 0.02 | -0.02 – 0.05 | 0.370 | -0.03 | -0.12 – 0.06 | 0.544 | -0.16 | -0.33 – 0.01 | 0.058 |
| Age | 0.01 | 0.01 – 0.01 | **<0.001** | -0.00 | -0.00 – 0.00 | 0.197 | 0.01 | 0.01 – 0.02 | **<0.001** | 0.00 | -0.00 – 0.01 | 0.394 |
| Wave 1 BMI | 0.00 | -0.00 – 0.01 | 0.061 | 0.00 | -0.00 – 0.00 | 0.869 | 0.00 | -0.00 – 0.01 | 0.190 | 0.01 | -0.00 – 0.02 | 0.152 |
| Race | -0.00 | -0.09 – 0.08 | 0.943 | -0.04 | -0.08 – 0.01 | 0.109 | -0.06 | -0.17 – 0.04 | 0.236 | 0.10 | -0.09 – 0.29 | 0.282 |
| Ethnicity | -0.05 | -0.15 – 0.05 | 0.320 | -0.03 | -0.08 – 0.02 | 0.179 | -0.02 | -0.15 – 0.10 | 0.716 | -0.01 | -0.22 – 0.20 | 0.955 |
| Education | -0.05 | -0.13 – 0.02 | 0.130 | 0.03 | -0.01 – 0.06 | 0.103 | 0.02 | -0.07 – 0.10 | 0.684 | -0.16 | -0.32 – -0.01 | **0.037** |
| LBP:CD14 × Gender | -0.04 | -0.08 – 0.01 | 0.091 | -0.01 | -0.03 – 0.02 | 0.588 | -0.04 | -0.10 – 0.02 | 0.207 | 0.03 | -0.07 – 0.12 | 0.573 |
| **Random Effects** | | | | | | | | | | | | |
| σ^2^ | 0.01 | | | 0.00 | | | 0.02 | | | 0.03 | | |
| τ_00_ | 0.05 _Id_ | | | 0.01 _Id_ | | | 0.07 _Id_ | | | 0.22 _Id_ | | |
| τ_11_ | 0.00 _Id.LBP_CD14_ | | | 0.00 _Id.LBP_CD14_ | | | 0.00 _Id.LBP_CD14_ | | | 0.01 _Id.LBP_CD14_ | | |
| ρ_01_ | 1.00 _Id_ | | | -0.42 _Id_ | | | -1.00 _Id_ | | | 0.60 _Id_ | | |
| ICC |  | | | 0.92 | | |  | | | 0.88 | | |
| N | 162 _Id_ | | | 162 _Id_ | | | 162 _Id_ | | | 162 _Id_ | | |
| Observations | 331 | | | 331 | | | 331 | | | 331 | | |
| Marginal R^2^ / Conditional R^2^ | 0.635 / NA | | | 0.069 / 0.922 | | | 0.673 / NA | | | 0.094 / 0.893 | | |

**Table S11: Prospective Model of Endotoxemia x Menopause for Laboratory Based Cognitive Tasks and Composite Working Memory Measure**

|  | **Composite Working Memory** | | | **Operation Span** | | | **Counting Span** | | | **Backwards Letter Span** | | | **AVLT** | | |
| --- | --- | --- | --- | --- | --- | --- | --- | --- | --- | --- | --- | --- | --- | --- | --- |
| *Predictors* | *Estimates* | *CI* | *p* | *Estimates* | *CI* | *p* | *Estimates* | *CI* | *p* | *Estimates* | *CI* | *p* | *Estimates* | *CI* | *p* |
| (Intercept) | 1.70 | 0.82 – 2.58 | **<0.001** | 18.67 | 11.10 – 26.25 | **<0.001** | 36.38 | 22.76 – 50.00 | **<0.001** | 23.03 | 15.87 – 30.19 | **<0.001** | 11.83 | 9.44 – 14.21 | **<0.001** |
| Time Linear | 0.14 | 0.04 – 0.24 | **0.007** | 0.36 | -0.75 – 1.48 | 0.521 | 2.90 | 0.72 – 5.08 | **0.009** | 0.75 | -0.19 – 1.68 | 0.117 | 0.25 | -0.09 – 0.59 | 0.154 |
| Time Quadratic | 0.03 | -0.07 – 0.12 | 0.595 | -0.75 | -1.83 – 0.33 | 0.171 | 2.02 | 0.13 – 3.90 | **0.036** | 0.06 | -0.86 – 0.98 | 0.898 | -0.35 | -0.69 – -0.01 | **0.041** |
| Wave 1 LBP:CD14 | 0.01 | -0.14 – 0.16 | 0.863 | 0.13 | -1.11 – 1.36 | 0.843 | -0.01 | -2.37 – 2.36 | 0.995 | 0.14 | -1.05 – 1.34 | 0.812 | 0.37 | -0.02 – 0.76 | 0.064 |
| Pre vs. Post Menopausal Women | 0.18 | -0.02 – 0.39 | 0.078 | 0.25 | -1.46 – 1.96 | 0.771 | 1.65 | -1.56 – 4.87 | 0.313 | 2.31 | 0.67 – 3.96 | **0.006** | 0.63 | 0.08 – 1.17 | **0.024** |
| Women vs. Men | 0.08 | -0.01 – 0.17 | 0.084 | 1.07 | 0.29 – 1.84 | **0.007** | 1.10 | -0.37 – 2.57 | 0.142 | 0.14 | -0.61 – 0.88 | 0.713 | -0.10 | -0.35 – 0.14 | 0.406 |
| Age | -0.03 | -0.04 – -0.02 | **<0.001** | -0.12 | -0.24 – 0.00 | 0.050 | -0.32 | -0.54 – -0.11 | **0.003** | -0.28 | -0.39 – -0.17 | **<0.001** | -0.05 | -0.09 – -0.02 | **0.004** |
| Wave 1 BMI | -0.00 | -0.02 – 0.01 | 0.625 | -0.06 | -0.20 – 0.08 | 0.400 | 0.01 | -0.25 – 0.27 | 0.929 | -0.02 | -0.15 – 0.12 | 0.801 | -0.02 | -0.06 – 0.03 | 0.415 |
| Race | -0.16 | -0.46 – 0.15 | 0.309 | -1.07 | -3.65 – 1.51 | 0.414 | -0.51 | -5.15 – 4.14 | 0.830 | -1.96 | -4.42 – 0.50 | 0.118 | -0.68 | -1.49 – 0.14 | 0.102 |
| Ethnicity | -0.18 | -0.52 – 0.16 | 0.307 | -1.95 | -4.89 – 1.00 | 0.194 | -3.26 | -8.50 – 1.99 | 0.222 | -0.30 | -3.08 – 2.48 | 0.833 | -0.43 | -1.36 – 0.50 | 0.359 |
| Education | 0.27 | 0.03 – 0.52 | **0.029** | 2.66 | 0.56 – 4.75 | **0.013** | 3.63 | -0.14 – 7.40 | 0.059 | 1.41 | -0.58 – 3.40 | 0.164 | 0.95 | 0.29 – 1.61 | **0.005** |
| Time Linear × Wave 1 LBP:CD14 | -0.12 | -0.22 – -0.01 | **0.025** | -0.60 | -1.75 – 0.56 | 0.309 | -2.15 | -4.39 – 0.08 | 0.059 | -0.68 | -1.64 – 0.27 | 0.161 | 0.23 | -0.12 – 0.58 | 0.205 |
| Time Quadratic × Wave 1 LBP:CD14 | 0.02 | -0.08 – 0.12 | 0.668 | -0.13 | -1.28 – 1.02 | 0.821 | 0.35 | -1.62 – 2.32 | 0.726 | 0.42 | -0.55 – 1.38 | 0.394 | -0.01 | -0.36 – 0.34 | 0.956 |
| Time Linear × Pre vs. Post Meno Women | -0.05 | -0.18 – 0.07 | 0.402 | -0.47 | -1.85 – 0.90 | 0.497 | -0.65 | -3.33 – 2.03 | 0.632 | -0.76 | -1.90 – 0.39 | 0.194 | 0.10 | -0.32 – 0.51 | 0.649 |
| Time Quadratic × Pre vs. Post Meno Women | -0.03 | -0.15 – 0.09 | 0.616 | -0.34 | -1.69 – 1.00 | 0.617 | 1.22 | -1.13 – 3.57 | 0.307 | -0.80 | -1.94 – 0.34 | 0.169 | 0.13 | -0.29 – 0.54 | 0.558 |
| Time Linear × Women vs. Men | 0.01 | -0.06 – 0.08 | 0.811 | -0.21 | -1.00 – 0.58 | 0.599 | 0.69 | -0.85 – 2.23 | 0.379 | -0.03 | -0.69 – 0.63 | 0.926 | 0.05 | -0.19 – 0.29 | 0.694 |
| Time Quadratic × Women vs. Men | -0.10 | -0.17 – -0.03 | **0.003** | -1.33 | -2.09 – -0.58 | **0.001** | -0.66 | -1.97 – 0.65 | 0.323 | -0.54 | -1.18 – 0.10 | 0.097 | 0.08 | -0.15 – 0.31 | 0.507 |
| Wave 1 LBP:CD14 × Pre vs. Post Meno Women | -0.02 | -0.18 – 0.15 | 0.838 | -0.36 | -1.72 – 1.00 | 0.602 | 0.23 | -2.35 – 2.82 | 0.858 | 0.21 | -1.10 – 1.52 | 0.752 | 0.10 | -0.32 – 0.53 | 0.632 |
| Wave 1 LBP:CD14 × Women vs. Men | 0.15 | 0.05 – 0.25 | **0.005** | 0.89 | 0.05 – 1.72 | **0.038** | 1.83 | 0.23 – 3.43 | **0.026** | 1.14 | 0.33 – 1.95 | **0.006** | 0.27 | 0.00 – 0.53 | **0.048** |
| Time Linear × Wave 1 LBP:CD14 ×  Pre vs. Post Meno Women | -0.02 | -0.14 – 0.09 | 0.696 | -0.74 | -2.06 – 0.59 | 0.273 | 0.21 | -2.38 – 2.81 | 0.873 | 0.07 | -1.04 – 1.17 | 0.907 | -0.26 | -0.67 – 0.15 | 0.210 |
| Time Quadratic × Wave 1 LBP:CD14 ×  Pre vs. Post Meno Women | 0.09 | -0.03 – 0.21 | 0.152 | 0.52 | -0.84 – 1.87 | 0.451 | 0.17 | -2.17 – 2.52 | 0.885 | 1.18 | 0.03 – 2.32 | **0.044** | 0.26 | -0.16 – 0.69 | 0.222 |
| Time Linear × Wave 1 LBP:CD14 ×  Women vs. Men | -0.02 | -0.09 – 0.06 | 0.680 | 0.04 | -0.83 – 0.90 | 0.933 | -0.16 | -1.82 – 1.51 | 0.852 | -0.06 | -0.77 – 0.65 | 0.863 | 0.02 | -0.24 – 0.28 | 0.861 |
| Time Quadratic × Wave 1 LBP:CD14 ×  Women vs. Men | -0.04 | -0.12 – 0.03 | 0.259 | -0.53 | -1.37 – 0.31 | 0.219 | -0.17 | -1.60 – 1.26 | 0.816 | -0.23 | -0.93 – 0.47 | 0.517 | -0.17 | -0.43 – 0.09 | 0.188 |
| **Random Effects** | | | | | | | | | | | | | | | |
| σ^2^ | 0.21 | | | 26.12 | | | 77.72 | | | 18.91 | | | 2.57 | | |
| τ_00_ | 0.50 _Id_ | | | 26.86 _Id_ | | | 103.71 _Id_ | | | 28.97 _Id_ | | | 2.80 _Id_ | | |
| τ_11_ | 0.00 _Id.burst_L1_ | | | 0.00 _Id.burst_L1_ | | | 25.87 _Id.burst_L1_ | | | 0.05 _Id.burst_L1_ | | | 0.01 _Id.burst_L1_ | | |
| ρ_01_ | 1.00 _Id_ | | | -1.00 _Id_ | | | 0.41 _Id_ | | | 1.00 _Id_ | | | -1.00 _Id_ | | |
| ICC |  | | |  | | | 0.57 | | |  | | |  | | |
| N | 162 _Id_ | | | 154 _Id_ | | | 160 _Id_ | | | 162 _Id_ | | | 162 _Id_ | | |
| Observations | 343 | | | 326 | | | 338 | | | 343 | | | 342 | | |
| Marginal R^2^ / Conditional R^2^ | 0.388 / NA | | | 0.241 / NA | | | 0.111 / 0.619 | | | 0.308 / NA | | | 0.232 / NA | | |

**Table S12: Prospective Model of Endotoxemia x Menopause for Ambulatory Cognitive Tasks**

|  | **Symbol Search** | | | **N-Back** | | | **N-Back (IES)** | | | **Spatial WM Error** | | |
| --- | --- | --- | --- | --- | --- | --- | --- | --- | --- | --- | --- | --- |
| *Predictors* | *Estimates* | *CI* | *p* | *Estimates* | *CI* | *p* | *Estimates* | *CI* | *p* | *Estimates* | *CI* | *p* |
| (Intercept) | 7.19 | 6.94 – 7.44 | **<0.001** | 0.99 | 0.87 – 1.11 | **<0.001** | 5.77 | 5.44 – 6.09 | **<0.001** | 0.69 | 0.15 – 1.23 | **0.012** |
| Time Linear | -0.06 | -0.08 – -0.04 | **<0.001** | 0.03 | 0.02 – 0.04 | **<0.001** | -0.23 | -0.26 – -0.20 | **<0.001** | -0.08 | -0.12 – -0.04 | **<0.001** |
| Time Quadratic | 0.01 | -0.01 – 0.03 | 0.218 | -0.01 | -0.01 – -0.00 | **0.017** | 0.05 | 0.02 – 0.08 | **0.001** | 0.04 | 0.01 – 0.07 | **0.017** |
| Wave 1 LBP:CD14 | -0.01 | -0.06 – 0.03 | 0.521 | -0.00 | -0.02 – 0.02 | 0.893 | -0.03 | -0.08 – 0.02 | 0.255 | -0.02 | -0.11 – 0.08 | 0.744 |
| Pre vs. Post Menopausal Women | -0.05 | -0.11 – 0.01 | 0.076 | 0.04 | 0.01 – 0.07 | **0.006** | -0.06 | -0.14 – 0.01 | 0.085 | -0.07 | -0.20 – 0.06 | 0.306 |
| Women vs. Men | 0.00 | -0.02 – 0.03 | 0.726 | 0.00 | -0.01 – 0.01 | 0.996 | 0.00 | -0.03 – 0.03 | 0.946 | -0.05 | -0.11 – 0.01 | 0.127 |
| Age | 0.01 | 0.01 – 0.02 | **<0.001** | -0.00 | -0.00 – -0.00 | **0.006** | 0.01 | 0.01 – 0.02 | **<0.001** | 0.01 | -0.00 – 0.01 | 0.142 |
| Wave 1 BMI | 0.00 | -0.00 – 0.01 | 0.077 | 0.00 | -0.00 – 0.00 | 0.843 | 0.00 | -0.00 – 0.01 | 0.212 | 0.01 | -0.00 – 0.02 | 0.187 |
| Race | -0.00 | -0.09 – 0.08 | 0.928 | -0.04 | -0.08 – 0.00 | 0.065 | -0.06 | -0.17 – 0.06 | 0.329 | 0.08 | -0.11 – 0.27 | 0.405 |
| Ethnicity | -0.06 | -0.16 – 0.04 | 0.227 | -0.03 | -0.07 – 0.02 | 0.285 | -0.04 | -0.17 – 0.09 | 0.538 | -0.04 | -0.25 – 0.17 | 0.696 |
| Education | -0.06 | -0.13 – 0.01 | 0.088 | 0.03 | -0.00 – 0.06 | 0.072 | 0.01 | -0.08 – 0.10 | 0.795 | -0.18 | -0.33 – -0.03 | **0.021** |
| Time Linear × Wave 1 LBP:CD14 | -0.00 | -0.02 – 0.02 | 0.873 | 0.00 | -0.01 – 0.01 | 0.812 | -0.02 | -0.05 – 0.02 | 0.328 | 0.02 | -0.02 – 0.07 | 0.327 |
| Time Quadratic × Wave 1 LBP:CD14 | 0.01 | -0.01 – 0.02 | 0.533 | -0.00 | -0.01 – 0.00 | 0.335 | 0.01 | -0.02 – 0.04 | 0.482 | 0.03 | -0.00 – 0.07 | 0.064 |
| Time Linear × Pre vs. Post Meno Women | 0.00 | -0.02 – 0.03 | 0.832 | 0.00 | -0.01 – 0.01 | 0.904 | -0.03 | -0.06 – 0.01 | 0.128 | 0.01 | -0.05 – 0.06 | 0.846 |
| Time Quadratic × Pre vs. Post Meno Women | -0.01 | -0.03 – 0.02 | 0.599 | -0.00 | -0.01 – 0.00 | 0.240 | 0.00 | -0.03 – 0.04 | 0.887 | 0.03 | -0.02 – 0.07 | 0.222 |
| Time Linear × Women vs. Men | -0.01 | -0.02 – 0.01 | 0.475 | -0.00 | -0.01 – 0.00 | 0.597 | -0.00 | -0.02 – 0.02 | 0.898 | 0.02 | -0.01 – 0.05 | 0.136 |
| Time Quadratic × Women vs. Men | -0.00 | -0.01 – 0.01 | 0.642 | 0.01 | 0.00 – 0.01 | **0.024** | -0.02 | -0.04 – -0.00 | **0.026** | -0.01 | -0.03 – 0.02 | 0.543 |
| Wave 1 LBP:CD14 × Pre vs. Post Meno Women | -0.01 | -0.05 – 0.04 | 0.799 | 0.00 | -0.02 – 0.02 | 0.964 | -0.01 | -0.07 – 0.05 | 0.716 | 0.03 | -0.08 – 0.13 | 0.595 |
| Wave 1 LBP:CD14 × Women vs. Men | -0.02 | -0.05 – 0.01 | 0.229 | 0.01 | -0.00 – 0.03 | 0.066 | -0.02 | -0.06 – 0.01 | 0.254 | -0.02 | -0.09 – 0.04 | 0.504 |
| Time Linear × Wave 1 LBP:CD14 ×  Pre vs. Post Meno Women | -0.01 | -0.03 – 0.01 | 0.465 | -0.00 | -0.01 – 0.01 | 0.796 | 0.01 | -0.02 – 0.05 | 0.419 | 0.06 | 0.00 – 0.11 | **0.035** |
| Time Quadratic × Wave 1 LBP:CD14 ×  Pre vs. Post Meno Women | -0.00 | -0.02 – 0.02 | 0.784 | -0.00 | -0.01 – 0.01 | 0.937 | 0.00 | -0.03 – 0.03 | 0.996 | 0.00 | -0.04 – 0.04 | 0.932 |
| Time Linear × Wave 1 LBP:CD14 ×  Women vs. Men | 0.01 | -0.01 – 0.02 | 0.447 | 0.00 | -0.00 – 0.01 | 0.467 | -0.01 | -0.04 – 0.01 | 0.303 | -0.00 | -0.04 – 0.03 | 0.777 |
| Time Quadratic ×Wave 1 LBP:CD14 ×  Women vs. Men | -0.00 | -0.02 – 0.01 | 0.553 | -0.00 | -0.01 – 0.00 | 0.415 | -0.00 | -0.02 – 0.02 | 0.944 | 0.02 | -0.00 – 0.05 | 0.061 |
| **Random Effects** | | | | | | | | | | | | |
| σ^2^ | 0.01 | | | 0.00 | | | 0.02 | | | 0.02 | | |
| τ_00_ | 0.05 _Id_ | | | 0.01 _Id_ | | | 0.07 _Id_ | | | 0.25 _Id_ | | |
| τ_11_ | 0.00 _Id.burst_L1_ | | | 0.00 _Id.burst_L1_ | | | 0.00 _Id.burst_L1_ | | | 0.02 _Id.burst_L1_ | | |
| ρ_01_ | 0.41 _Id_ | | | -0.33 _Id_ | | | -0.27 _Id_ | | | 0.57 _Id_ | | |
| ICC | 0.87 | | | 0.92 | | | 0.80 | | | 0.91 | | |
| N | 162 _Id_ | | | 162 _Id_ | | | 162 _Id_ | | | 162 _Id_ | | |
| Observations | 355 | | | 355 | | | 355 | | | 355 | | |
| Marginal R^2^ / Conditional R^2^ | 0.218 / 0.902 | | | 0.123 / 0.930 | | | 0.311 / 0.863 | | | 0.094 / 0.920 | | |

**Table S13: Coupling Model of Endotoxemia x Menopause for Laboratory Cognitive Tasks and Composite Working Memory**

|  | **Composite Working Memory** | | | **Operation Span** | | | **Counting Span** | | | **Backwards Letter Span** | | | **AVLT** | | |
| --- | --- | --- | --- | --- | --- | --- | --- | --- | --- | --- | --- | --- | --- | --- | --- |
| *Predictors* | *Estimates* | *CI* | *p* | *Estimates* | *CI* | *p* | *Estimates* | *CI* | *p* | *Estimates* | *CI* | *p* | *Estimates* | *CI* | *p* |
| (Intercept) | 1.68 | 0.82 – 2.54 | **<0.001** | 18.78 | 11.59 – 25.97 | **<0.001** | 36.23 | 22.79 – 49.66 | **<0.001** | 23.06 | 15.98 – 30.14 | **<0.001** | 11.46 | 9.10 – 13.81 | **<0.001** |
| Time Linear | 0.15 | 0.03 – 0.27 | **0.014** | 0.66 | -0.60 – 1.93 | 0.304 | 2.67 | 0.35 – 4.98 | **0.024** | 0.83 | -0.19 – 1.86 | 0.111 | 0.20 | -0.19 – 0.59 | 0.319 |
| Time Quadratic | 0.04 | -0.06 – 0.14 | 0.440 | -0.53 | -1.65 – 0.59 | 0.355 | 1.93 | -0.09 – 3.95 | 0.061 | 0.17 | -0.69 – 1.02 | 0.701 | -0.37 | -0.71 – -0.03 | **0.035** |
| LBP:CD14 | 0.01 | -0.11 – 0.12 | 0.894 | -0.15 | -1.22 – 0.93 | 0.790 | 0.36 | -1.72 – 2.44 | 0.735 | 0.10 | -0.90 – 1.10 | 0.848 | 0.03 | -0.31 – 0.38 | 0.848 |
| Pre vs. Post Menopausal Women | 0.18 | -0.02 – 0.38 | 0.074 | 0.02 | -1.58 – 1.62 | 0.982 | 1.99 | -1.08 – 5.05 | 0.203 | 2.22 | 0.59 – 3.85 | **0.008** | 0.57 | 0.04 – 1.11 | **0.036** |
| Women vs. Men | 0.08 | -0.01 – 0.17 | 0.082 | 1.09 | 0.37 – 1.81 | **0.003** | 1.01 | -0.35 – 2.38 | 0.145 | 0.15 | -0.58 – 0.88 | 0.681 | -0.09 | -0.32 – 0.15 | 0.465 |
| Age | -0.03 | -0.04 – -0.02 | **<0.001** | -0.10 | -0.21 – 0.01 | 0.078 | -0.31 | -0.52 – -0.10 | **0.004** | -0.27 | -0.39 – -0.16 | **<0.001** | -0.05 | -0.09 – -0.02 | **0.006** |
| Wave 1 BMI | -0.00 | -0.02 – 0.01 | 0.573 | -0.07 | -0.19 – 0.06 | 0.295 | -0.02 | -0.28 – 0.23 | 0.858 | -0.03 | -0.16 – 0.10 | 0.656 | -0.01 | -0.05 – 0.04 | 0.722 |
| Race | -0.15 | -0.45 – 0.15 | 0.323 | -1.88 | -4.28 – 0.52 | 0.125 | 0.22 | -4.36 – 4.79 | 0.926 | -1.83 | -4.29 – 0.63 | 0.144 | -0.79 | -1.60 – 0.02 | 0.056 |
| Ethnicity | -0.17 | -0.50 – 0.17 | 0.333 | -2.69 | -5.50 – 0.12 | 0.061 | -2.15 | -7.31 – 3.02 | 0.414 | -0.22 | -2.98 – 2.54 | 0.875 | -0.60 | -1.53 – 0.34 | 0.209 |
| Education | 0.27 | 0.03 – 0.51 | **0.028** | 2.72 | 0.76 – 4.67 | **0.007** | 3.30 | -0.41 – 7.01 | 0.081 | 1.45 | -0.53 – 3.43 | 0.151 | 0.89 | 0.24 – 1.54 | **0.007** |
| LBP:CD14 × Pre vs. Post Menopausal Women | -0.07 | -0.18 – 0.04 | 0.217 | -0.36 | -1.40 – 0.68 | 0.499 | -1.02 | -3.02 – 0.98 | 0.314 | -0.48 | -1.44 – 0.47 | 0.318 | 0.06 | -0.27 – 0.38 | 0.735 |
| LBP:CD14 × Women vs. Men | 0.12 | 0.05 – 0.19 | **0.001** | 0.90 | 0.23 – 1.57 | **0.009** | 1.72 | 0.47 – 2.98 | **0.007** | 0.90 | 0.31 – 1.50 | **0.003** | 0.10 | -0.11 – 0.30 | 0.371 |
| **Random Effects** | | | | | | | | | | | | | | | |
| σ^2^ | 0.22 | | | 28.05 | | | 90.91 | | | 16.20 | | | 2.66 | | |
| τ_00_ | 0.45 _Id_ | | | 20.90 _Id_ | | | 84.94 _Id_ | | | 30.07 _Id_ | | | 2.68 _Id_ | | |
| τ_11_ | 0.00 _Id.LBP_CD14_ | | | 2.31 _Id.LBP_CD14_ | | | 0.38 _Id.LBP_CD14_ | | | 0.10 _Id.LBP_CD14_ | | | 0.01 _Id.LBP_CD14_ | | |
| ρ_01_ | 1.00 _Id_ | | | -0.77 _Id_ | | | 1.00 _Id_ | | | 1.00 _Id_ | | | -1.00 _Id_ | | |
| ICC |  | | | 0.45 | | |  | | |  | | |  | | |
| N | 162 _Id_ | | | 154 _Id_ | | | 160 _Id_ | | | 162 _Id_ | | | 161 _Id_ | | |
| Observations | 330 | | | 313 | | | 325 | | | 330 | | | 329 | | |
| Marginal R^2^ / Conditional R^2^ | 0.345 / NA | | | 0.130 / 0.523 | | | 0.169 / NA | | | 0.309 / NA | | | 0.170 / NA | | |

**Table S14: Coupling Model of Endotoxemia x Menopause for Ambulatory Cognitive Tasks**

|  | **Symbol Search** | | | **N-Back** | | | **N-Back (IES)** | | | **Spatial WM Error** | | |
| --- | --- | --- | --- | --- | --- | --- | --- | --- | --- | --- | --- | --- |
| *Predictors* | *Estimates* | *CI* | *p* | *Estimates* | *CI* | *p* | *Estimates* | *CI* | *p* | *Estimates* | *CI* | *p* |
| (Intercept) | 7.20 | 6.96 – 7.45 | **<0.001** | 0.99 | 0.87 – 1.12 | **<0.001** | 5.71 | 5.40 – 6.02 | **<0.001** | 0.69 | 0.15 – 1.23 | **0.012** |
| Time Linear | -0.06 | -0.09 – -0.04 | **<0.001** | 0.02 | 0.01 – 0.03 | **<0.001** | -0.22 | -0.26 – -0.19 | **<0.001** | -0.10 | -0.15 – -0.05 | **<0.001** |
| Time Quadratic | 0.01 | -0.01 – 0.03 | 0.246 | -0.01 | -0.02 – -0.00 | **0.033** | 0.05 | 0.02 – 0.08 | **0.001** | 0.04 | 0.00 – 0.08 | **0.049** |
| LBP:CD14 | 0.00 | -0.03 – 0.03 | 0.965 | 0.01 | -0.00 – 0.02 | 0.200 | -0.02 | -0.06 – 0.01 | 0.194 | 0.02 | -0.04 – 0.08 | 0.478 |
| Pre vs. Post Menopausal Women | -0.05 | -0.11 – 0.01 | 0.075 | 0.04 | 0.01 – 0.07 | **0.005** | -0.06 | -0.13 – 0.01 | 0.079 | -0.05 | -0.17 – 0.08 | 0.468 |
| Women vs. Men | 0.00 | -0.02 – 0.03 | 0.759 | 0.00 | -0.01 – 0.01 | 0.826 | -0.00 | -0.03 – 0.03 | 0.854 | -0.05 | -0.11 – 0.01 | 0.090 |
| Age | 0.01 | 0.01 – 0.01 | **<0.001** | -0.00 | -0.00 – -0.00 | **0.006** | 0.01 | 0.01 – 0.02 | **<0.001** | 0.01 | -0.00 – 0.01 | 0.223 |
| Wave 1 BMI | 0.00 | -0.00 – 0.01 | 0.072 | 0.00 | -0.00 – 0.00 | 0.893 | 0.00 | -0.00 – 0.01 | 0.171 | 0.01 | -0.00 – 0.02 | 0.163 |
| Race | 0.00 | -0.09 – 0.09 | 0.980 | -0.04 | -0.08 – 0.00 | 0.060 | -0.06 | -0.16 – 0.05 | 0.301 | 0.11 | -0.08 – 0.30 | 0.257 |
| Ethnicity | -0.06 | -0.16 – 0.04 | 0.237 | -0.03 | -0.08 – 0.02 | 0.271 | -0.03 | -0.16 – 0.09 | 0.594 | -0.01 | -0.23 – 0.20 | 0.894 |
| Educationc | -0.06 | -0.13 – 0.01 | 0.112 | 0.03 | -0.00 – 0.06 | 0.085 | 0.02 | -0.07 – 0.10 | 0.682 | -0.16 | -0.32 – -0.01 | **0.035** |
| LBP:CD14 × Pre vs. Post Menopausal Women | 0.01 | -0.02 – 0.03 | 0.463 | 0.00 | -0.01 – 0.01 | 0.946 | 0.00 | -0.03 – 0.03 | 0.971 | 0.02 | -0.04 – 0.08 | 0.505 |
| LBP:CD14 × Women vs. Men | -0.01 | -0.03 – 0.00 | 0.066 | -0.00 | -0.01 – 0.01 | 0.635 | -0.01 | -0.03 – 0.01 | 0.210 | 0.01 | -0.03 – 0.04 | 0.695 |
| **Random Effects** | | | | | | | | | | | | |
| σ^2^ | 0.01 | | | 0.00 | | | 0.02 | | | 0.03 | | |
| τ_00_ | 0.05 _Id_ | | | 0.01 _Id_ | | | 0.06 _Id_ | | | 0.23 _Id_ | | |
| τ_11_ | 0.00 _Id.LBP_CD14_ | | | 0.00 _Id.LBP_CD14_ | | | 0.00 _Id.LBP_CD14_ | | | 0.01 _Id.LBP_CD14_ | | |
| ρ_01_ | 1.00 _Id_ | | | -0.41 _Id_ | | | -0.93 _Id_ | | | 0.58 _Id_ | | |
| ICC |  | | | 0.91 | | | 0.78 | | | 0.88 | | |
| N | 162 _Id_ | | | 162 _Id_ | | | 162 _Id_ | | | 162 _Id_ | | |
| Observations | 331 | | | 331 | | | 331 | | | 331 | | |
| Marginal R^2^ / Conditional R^2^ | 0.655 / NA | | | 0.113 / 0.924 | | | 0.323 / 0.848 | | | 0.099 / 0.894 | | |

**Table S15: Prospective Model of Endotoxemia x Age for Composite Working Memory**

|  | **Composite Working Memory** | | |
| --- | --- | --- | --- |
| *Predictors* | *Estimates* | *CI* | *p* |
| (Intercept) | 1.22 | 0.40 – 2.04 | **0.004** |
| Time Linear | 0.37 | -0.07 – 0.80 | 0.098 |
| Time Quadratic | 0.23 | -0.17 – 0.62 | 0.259 |
| Wave 1 LBP:CD14 | -0.22 | -0.74 – 0.30 | 0.403 |
| Age | -0.02 | -0.03 – -0.01 | **0.001** |
| Wave 1 BMI | -0.00 | -0.02 – 0.01 | 0.574 |
| Gender | 0.23 | -0.03 – 0.50 | 0.088 |
| Race | -0.13 | -0.45 – 0.18 | 0.403 |
| Ethnicity | -0.24 | -0.59 – 0.11 | 0.182 |
| Education | 0.23 | -0.02 – 0.49 | 0.068 |
| Time Linear × Wave 1 LBP:CD14 | -0.19 | -0.58 – 0.19 | 0.332 |
| Time Quadratic × Wave 1 LBP:CD14 | 0.09 | -0.28 – 0.46 | 0.641 |
| Time Linear × Age | -0.00 | -0.01 – 0.00 | 0.325 |
| Time Quadratic × Age | -0.00 | -0.01 – 0.00 | 0.316 |
| Wave 1 LBP:CD14 × Age | 0.00 | -0.01 – 0.02 | 0.448 |
| (Time Linear × Wave 1 LBP:CD14) × Age | 0.00 | -0.01 – 0.01 | 0.664 |
| (Time Quadratic × Wave 1 LBP:CD14) × Age | -0.00 | -0.01 – 0.01 | 0.658 |
| **Random Effects** | | | |
| σ^2^ | 0.22 | | |
| τ_00_ _Id_ | 0.53 | | |
| τ_11_ _Id.burst_L1_ | 0.00 | | |
| ρ_01_ _Id_ | 1.00 | | |
| N _Id_ | 162 | | |
| Observations | 343 | | |
| Marginal R^2^ / Conditional R^2^ | 0.277 / NA | | |

**Table S16: Coupling Model of Endotoxemia x Age for Composite Working Memory**

|  | **Composite Working Memory** | | |
| --- | --- | --- | --- |
| *Predictors* | *Estimates* | *CI* | *p* |
| (Intercept) | 1.20 | 0.41 – 1.99 | **0.003** |
| Time Linear | 0.16 | 0.04 – 0.28 | **0.009** |
| Time Quadratic | 0.03 | -0.07 – 0.13 | 0.589 |
| LBP:CD14 | -0.11 | -0.48 – 0.26 | 0.568 |
| Age | -0.02 | -0.03 – -0.01 | **0.001** |
| Wave 1 BMI | -0.01 | -0.02 – 0.01 | 0.487 |
| Gender | 0.21 | -0.05 – 0.48 | 0.114 |
| Race | -0.13 | -0.44 – 0.18 | 0.416 |
| Ethnicity | -0.22 | -0.57 – 0.13 | 0.210 |
| Education | 0.24 | -0.00 – 0.49 | 0.054 |
| LBP:CD14 × Age | 0.00 | -0.01 – 0.01 | 0.603 |
| **Random Effects** | | | |
| σ^2^ | 0.22 | | |
| τ_00_ _Id_ | 0.49 | | |
| τ_11_ _Id.LBP_CD14_ | 0.01 | | |
| ρ_01_ _Id_ | 0.09 | | |
| ICC | 0.70 | | |
| N _Id_ | 162 | | |
| Observations | 330 | | |
| Marginal R^2^ / Conditional R^2^ |  |  |  |

**Table S17: Wave 1 Cross-Sectional Analysis of Endotoxemia x Gender**

|  | **Composite Working Memory** | | |
| --- | --- | --- | --- |
| *Predictors* | *Estimates* | *CI* | *p* |
| (Intercept) | 0.93 | 0.10 – 1.76 | **0.028** |
| Wave 1 LBP:CD14 | -0.07 | -0.23 – 0.09 | 0.372 |
| Gender | 0.18 | -0.09 – 0.46 | 0.194 |
| Wave 1 BMI | 0.00 | -0.02 – 0.02 | 0.929 |
| Age | -0.02 | -0.03 – -0.01 | **0.001** |
| Race | -0.09 | -0.42 – 0.23 | 0.574 |
| Ethnicity | -0.15 | -0.52 – 0.21 | 0.403 |
| Education | 0.24 | -0.02 – 0.50 | 0.076 |
| Wave 1 LBP:CD14 × Gender | 0.42 | 0.12 – 0.72 | **0.006** |
| Observations | 161 | | |
| R^2^ / R^2^ adjusted | 0.126 / 0.080 | | |

**Supplemental References**

Bake, S., Friedman, J. A., & Sohrabji, F. (2009). Reproductive age-related changes in the blood

brain barrier: expression of IgG and tight junction proteins. Microvasc Res. 78(3). https://doi.org/10.1016/j.mvr.2009.06.009

Cipolla, M. J., Godfrey, J. A., & Wiegman, M. J. (2009). The effect of ovariectomy and estrogen

on penetrating brain arterioles and blood-brain barrier permeability. Microcirculation. 16(8). <https://doi.org/10.3109/10739680903164131>

Wilson, A. C., Clemente, L., Liu, T., Bowen, R. L., Meethal, S. V., & Atwood, C. S.

(2008). Reproductive hormones regulate the selective permeability of the blood-

brain barrier. Biochim Biophys Acta. 1782(6). https://doi.org/10.1016/j.bbadis.2008.02.011
